# Fully bio-based composite and modular metastructures

**DOI:** 10.1007/s42114-025-01359-1

**Published:** 2025-07-01

**Authors:** Rodrigo José da Silva, Bárbara Lana de Resende, Gianni Comandini, Jacopo Lavazza, Pedro P. Camanho, Fabrizio Scarpa, Túlio Hallak Panzera

**Affiliations:** 1 CERN, the European Organization for Nuclear Research, R&D Programme EP-DT, Geneva, Switzerland; 2 Centre for Innovation and Technology in Composite Materials (CITeC), Federal University of São João del-Rei (UFSJ), São João del-Rei, Brazil; 3 Bristol Composites Institute, School of Civil, Aerospace and Design Engineering (CADE), University of Bristol, Bristol, UK; 4 INEGI, Faculdade de Engenharia, Universidade Do Porto, Porto, Portugal

**Keywords:** Composite structure, Metastructure, Truss, Lattice, Bamboo, Beam, Sandwich structure, Bio-based polymers

## Abstract

The reliance on fossil-derived components in the design of metamaterials and metastructures presents sustainability and environmental challenges, prompting the development of alternative solutions. In response, this study proposes a fully bio-based and modular metastructure composed of rods extracted from the giant bamboo (*Dendrocalamus asper*) and plant-based polymeric joints derived from soybean (*Glycine max*) and castor oil (*Ricinus communis*), aiming to offer a sustainable alternative for load-bearing structural components. The research investigates the design, fabrication, and mechanical performance of a unit trussed cell (50 × 50 × 50 mm^3^) engineered to exhibit auxetic-like chiral rotation and enhanced energy absorption under compressive loading. These cells are assembled into trussed beams (400 × 50 × 50 mm^3^), and further into sandwich beams with 5 mm thick balsa wood skins. Material properties of the bamboo and polymer components are assessed via physical, chemical, and mechanical characterisation to asses their potential chemical-adhesion compatibility, density, and mechanical performance. Following the fabrication of the proposed structures, further experimental evaluation includes compression of the trussed cell and four-point bending of the beam configurations, while finite element analysis (FEA) is used to simulate elastic behaviour under torsional and cantilever loading. Results demonstrate that the metastructure trussed cell (with a mass of ~ 30 g) supports up to 700 kg in compression, achieving ~ 2 mm displacement, 4° rotation, and absorbing ~ 750 μJ/mm^3^ of energy; it also exhibits a force–displacement slope of ~ 4,200 N/mm and an equivalent Poisson ratio near zero within the elastic regime (up to ~ 1 mm displacement). The trussed and sandwich beams exhibit equivalent densities of ~ 0.19 and ~ 0.21 g/cm^3^, respectively, while achieving bending loads of ~ 2000 N and ~ 3600 N, corresponding to maximum bending moments of ~ 103 and ~ 188 kN∙mm, and toughness values of ~ 158 and ~ 193 μJ/mm^3^, respectively. Simulated torsional response of the trussed cell indicates a torque of ~ 7,300 N∙mm per degree of twist, while FEA results for cantilever loading show a homogenised flexural modulus of the beams of ~ 623 MPa (trussed) and ~ 751 MPa (sandwich). These outcomes underscore a promising direction for developing renewable, high-strength, and lightweight composite structures, with applications ranging from civil construction to aerospace engineering.

**Graphical Abstract:**

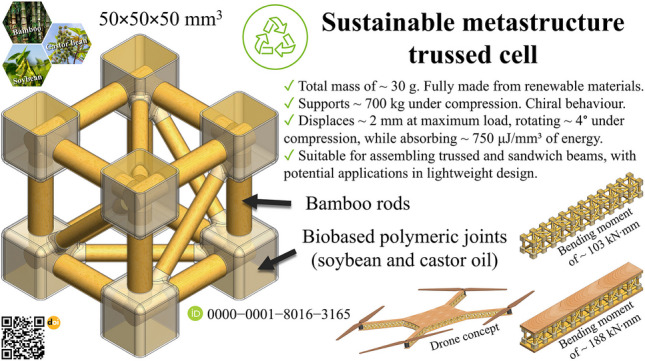

## Introduction

Metamaterials and metastructures are engineered to achieve specific mechanical properties by controlling their internal architecture, enabling enhanced stiffness, strength-to-weight ratio, and energy absorption. Their structural configurations, including trussed lattices [1] and sandwich designs [2], allow for precise mechanical performance tuning beyond the limitations of conventional materials. These architected systems offer unique opportunities for tailoring mechanical responses through geometry, rather than solely relying on material composition. Although traditionally manufactured using fossil-based resources, increasing emphasis is being placed on the use of renewable and recyclable constituents [3]. As engineering moves toward greener practices and circular economy principles, the integration of natural and bio-based materials into high-performance structures has become a key research frontier. In this context, the present manuscript introduces a novel approach to sustainable metastructures, proposing a modular cell concept based on bio-based materials and designed for efficient mechanical performance in load-bearing applications, further incorporating trussed and sandwich structures.

**Fig. 1:**
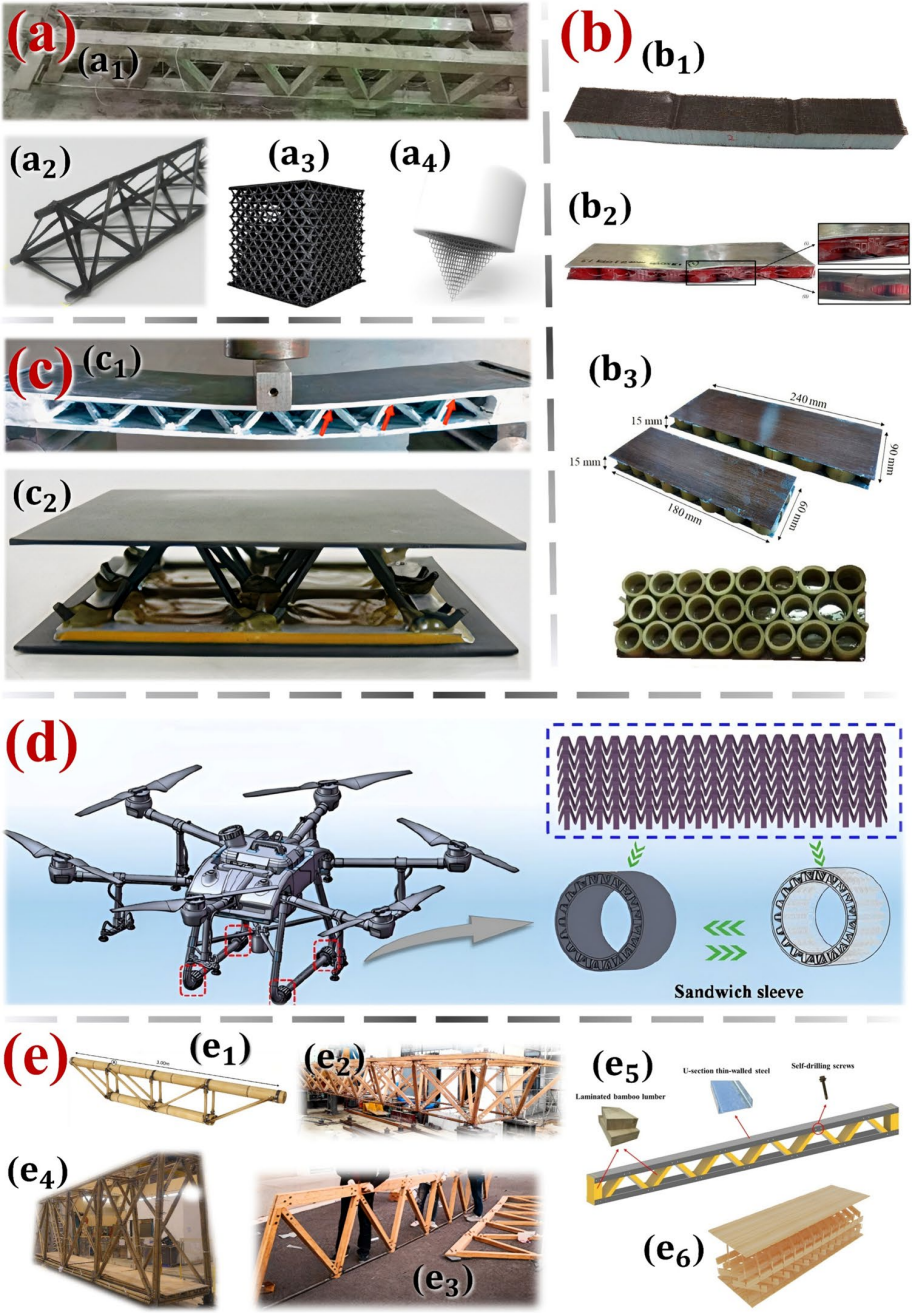
**a** structural applications of truss-based geometries, **b** sustainable structures based on eco-friendly materials, **c** sandwich panels with a lattice core, **d** metastructure lattice core for sandwich design, and **e** truss structures made of bamboo (whole or laminated culms)

Starting from trussed structures, which are distinguished by their ability to sustain substantial loads with minimal material usage, these systems rely on geometric configurations made by members interconnected at joints. Historically fabricated from wood, steel, or a combination thereof, trusses have been extensively employed in applications ranging from buildings and bridges to towers. In modern materials science and engineering, truss-based or lattice geometries transcend traditional scales and functions, appearing in diverse domains: from micro to macro scales, composite materials to auxetic structures, and secondary applications to high-performance systems. Figure 1a depicts some cases of engineering designs based on truss geometries: in Fig. 1a_1_, tubular steel trusses proposed for civil construction, filled with cementitious composite reinforced with metallic fibres [4]; in Fig. 1a_2_, high-performance applications for dimensions of the order of tens of millimetres [5], employing trusses made of carbon fibres through a filament winding process; in Fig. 1a_3_, carbon composite truss structures produced via additive manufacturing [6]; and, in Fig. 1a_4_, a cellular microstructure with lattice geometry to replace conventional atomic force microscopy tips [7].

**Fig. 2:**
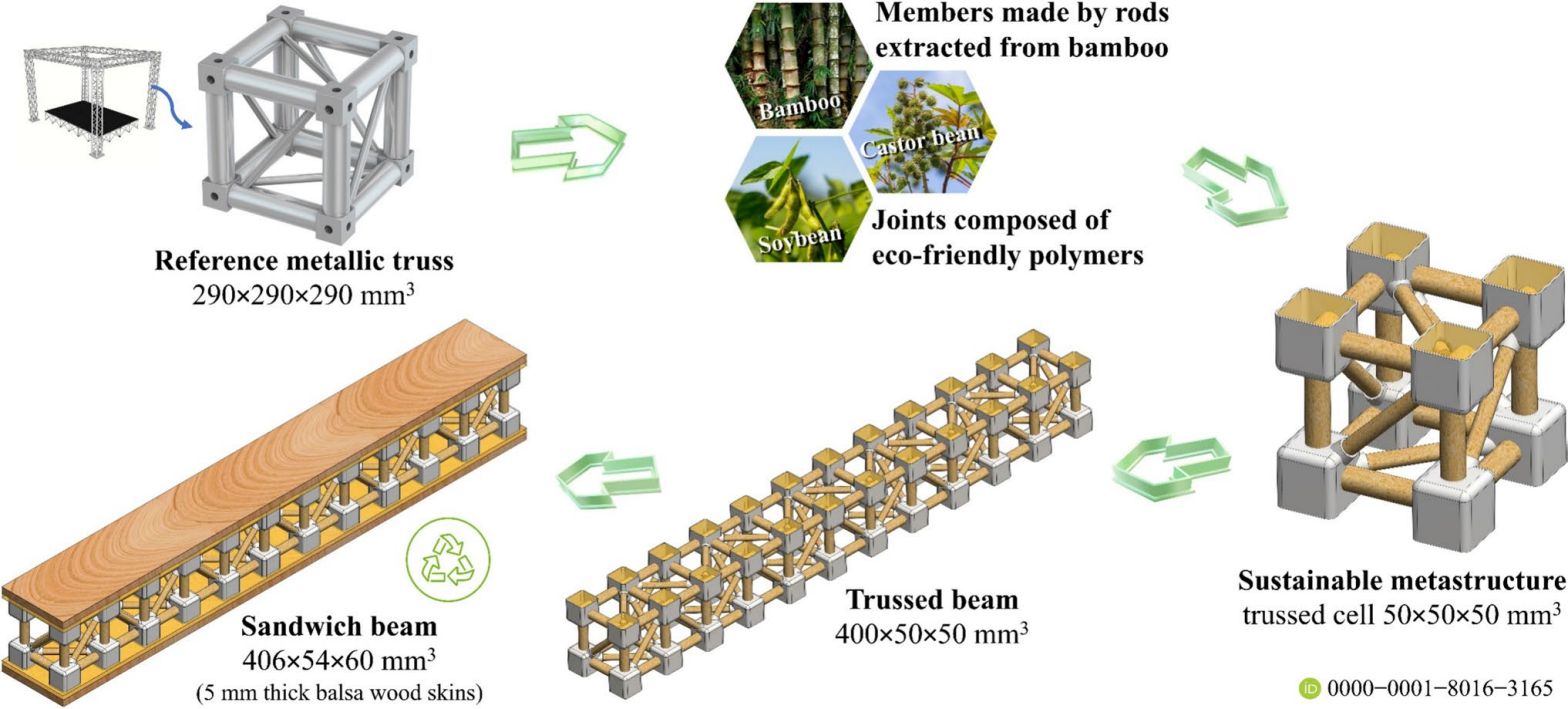
Concept of the proposed metastructure cell composed of eco-friendly materials and its application for modular assembly of trussed and sandwich beams

**Fig. 3:**
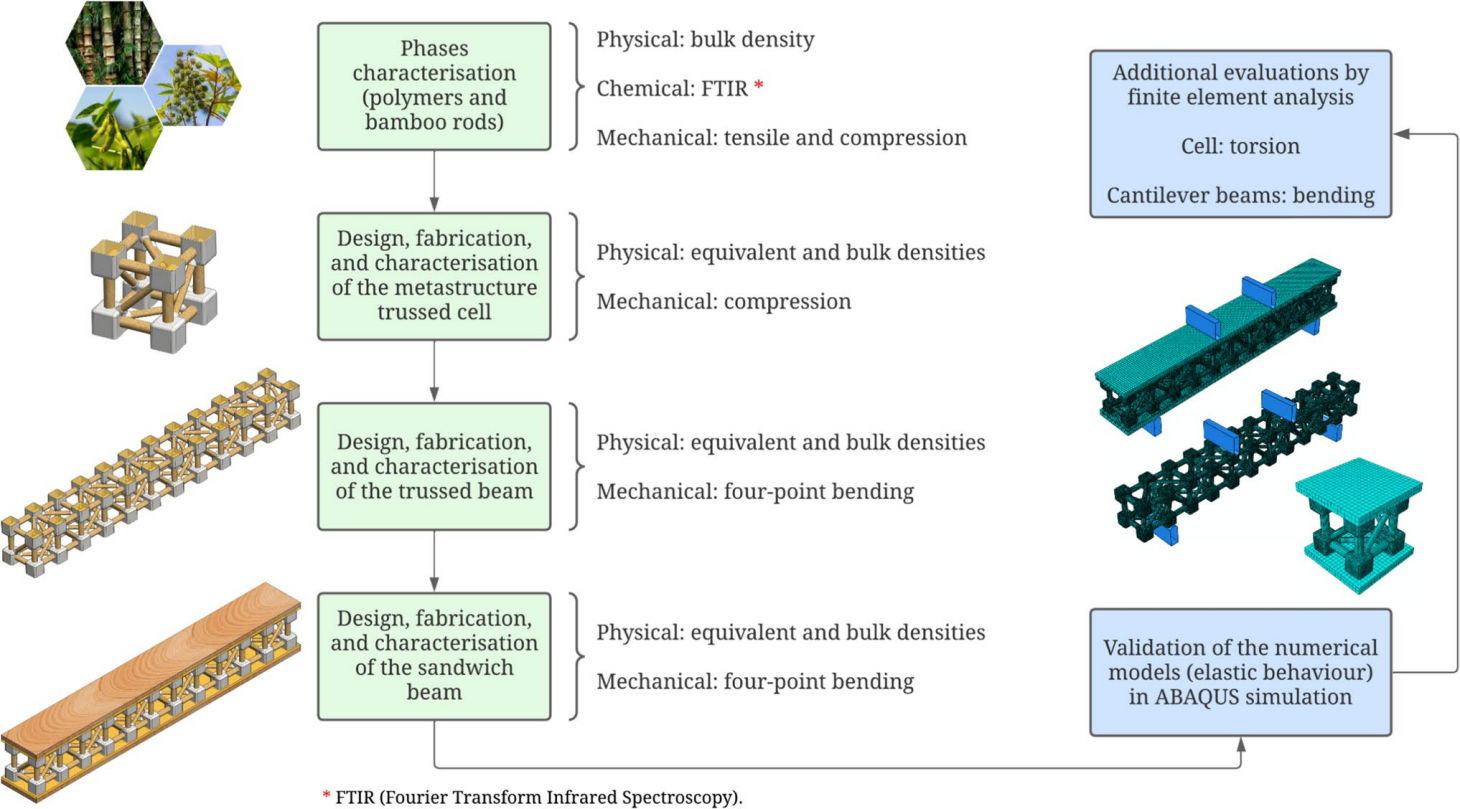
Flowchart detailing the research activities carried out in this work

**Fig. 4:**
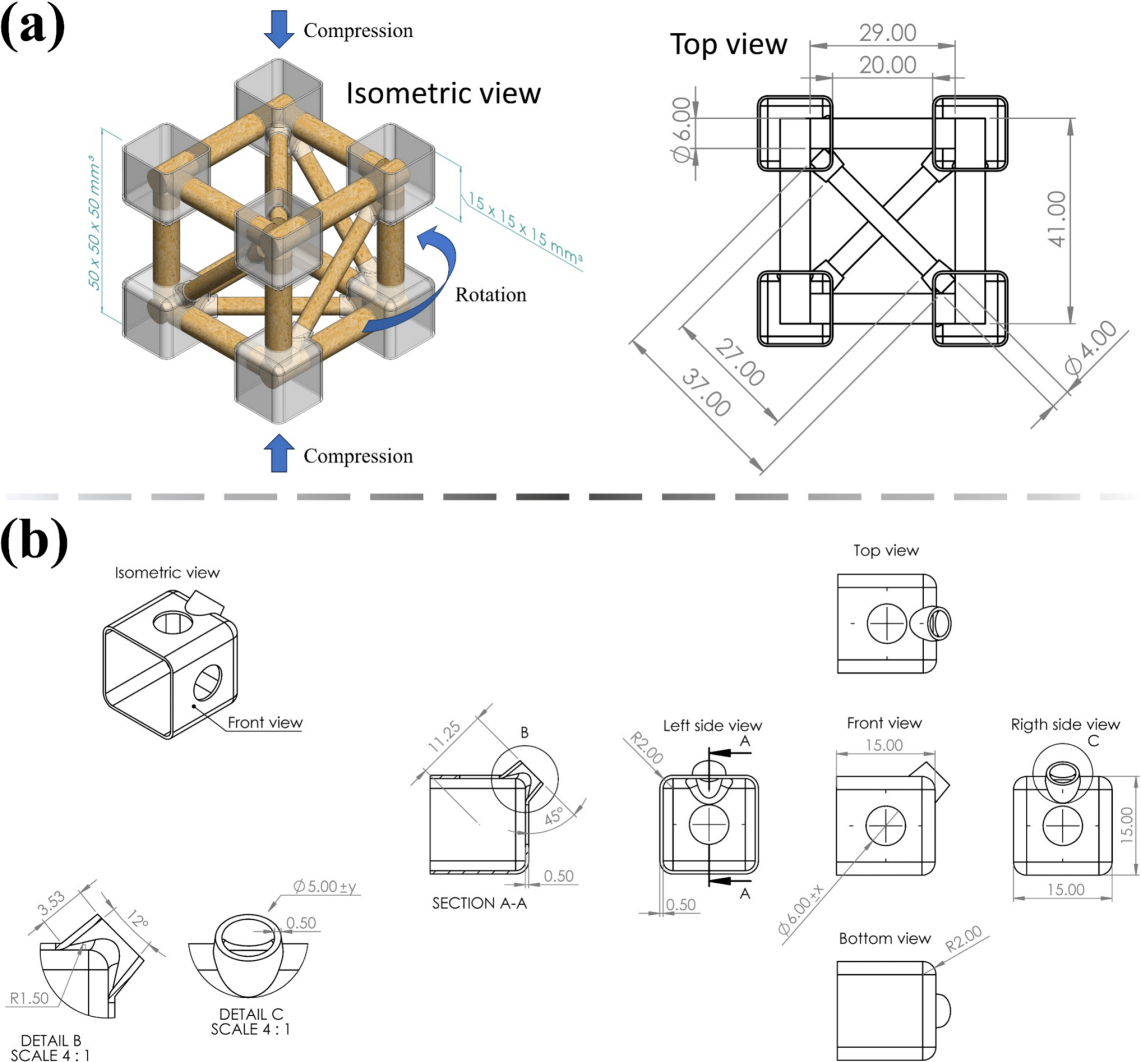
Sustainable composite design: **a** metastructure trussed cell and **b** shell that compounds the joints (dimensions in mm)

On the subject of research linked to sustainability, there is a growing tendency to aim at the development of new products with characteristics that make them more efficient in terms of energy, recycling, and biodegradability [8–15]. Concerning sustainable sandwich structural components, recent examples are the ones made of composites in hemp fibres and recycled PET foam [13], in Fig. 1b_1_; the sandwich structures with a core of PET bottle caps [14], in Fig. 1b_2_; or even panels with prepreg flax skins and bamboo rings core [15], in Fig. 1b_3_. Resembling truss structures, the sandwich panels are distinguished by their higher strength-to-weight ratio, but specifically when subjected to bending efforts. This characteristic is intrinsic to sandwich materials in general, in which the thin faces (also denoted by skins or facesheets) provide most of the resistance, whilst the less-density and less-rigid core material aims to add thickness to the structure, to provide an increase in the moment of inertia and consequent enhancement in the overall stiffness. The possibility of combining sandwich panels with a truss-based core is well established and widely discussed in recent decades [16–23] – refer to cases in point in Fig. 1c.

As initially contextualised, metastructures are designed through advanced architectures with tailored properties, such as negative Poisson ratio, which are not commonly found in conventional structures [24]. Metastructures can exhibit various geometric configurations, including the aforementioned truss-based or lattice frameworks [25]. By precisely adjusting the geometry and spatial arrangement of their constituent elements, metastructures can achieve functionality beyond the intrinsic limitations of base structures, enabling innovations in a range of sectors, such as aerospace [26] and civil construction [27]. Metastructures with lattice-based geometry can also be applied in sandwich components, offering potential enhancements in mechanical performance and multifunctional capabilities, such as vibrance reduction [28]. A case in point of a lattice metastructure concept employed as the core in a sandwich design is illustrated in Fig. 1d, which serves as a cushioning protection device for the bracket of drones [29].

### General research aims

Leveraging the versatility of lattice metastructures and addressing global concerns regarding clean production and waste minimisation, this research focuses on the design, fabrication, characterisation, and application of a trussed metastructure cell composed of eco-friendly materials – Fig. 2. The design draws inspiration from cubic metal trusses, widely commercialised for stage setups at events. The sustainable cells feature members made of rods extracted from bamboo culms and biphasic joints comprising two environmentally friendly polymers: (i) an external shell derived from soybean oil and (ii) a filling synthesised from castor oil. The modular assembly of the composite trussed cell is engineered to rotate under compression, making it a metastructure with the potential for energy absorption under quasi-static loading. Trussed beams are constructed through serial integration of these cells, ensuring scalability for diverse structural applications. Their use as the core of sandwich beams is proposed by bonding balsa wood skins with the castor oil polymer as an adhesive system, in an attempt to enhance the load-bearing capacity and overall mechanical performance. This innovative design, combining natural materials and sustainable polymers, results in a fully renewable composite structure that exemplifies environmental stewardship and advanced composite design.

Bamboo (*Bambusoideae*) is one of the fastest-growing plant species, with three to five years of maturation [30]. Bamboo is a renewable source of raw material, being efficient in absorbing carbon dioxide from the atmosphere and presents a potential contribution to the reduction of greenhouse gases [31]. Furthermore, bamboo is a naturally optimised composite material [32], exhibiting a hierarchical structure that enhances its strength-to-weight ratio. Its unique arrangement of cellulose fibres within a lignin matrix enhances mechanical properties, enabling it to withstand both compressive and tensile stresses. This natural design, optimised through evolution, results in high load-bearing capacity and resilience, making bamboo an excellent resource for sustainable material development and bio-inspired engineering applications [30–33]. Natural composite rods can be extracted from the culms of the bamboo [34], therefore basing the choice of bamboo rods as the raw material to be used as the members of the here-proposed trussed metastructure.

Soybean (*Glycine max*) and castor bean (*Ricinus communis*) are high-yielding, low-input crops that mature within 2–5 months, require minimal resources, and demonstrate strong adaptability to various soils and adverse climates; characteristics which offer a reduced environmental impact compared to other species [35, 36]. The oils extracted from both plants possess a high content of unsaturated fatty acids and hydroxyl groups, which provide reactive sites for chemical modifications, enabling the synthesis of versatile polymeric compounds with tailored mechanical and thermal properties [37, 38]. In this context, the polymers derived from both plants demonstrate strong suitability for the fabrication of the joints in the presently designed trussed metastructure.

Originating in tropical regions, the balsa tree (*Ochroma pyramidale*) is notable for its combination of mechanical performance, low weight, and sustainability [39]. Balsa contributes to atmospheric carbon sequestration, and its wood is recyclable [40], which makes it highly suitable for engineering design. Common applications include civil construction, nautical components, and the model aircraft industry, in which balsa wood is typically employed as the core material in sandwich structures [39–41]. Despite this tendency to apply balsa wood as the core of sandwich panels, it is important to emphasise that the intrinsic high strength-to-weight ratio of this natural material supports the research proposal to explore its implementation as the skin layers of the sustainable sandwich beam, as depicted in Fig. 2.

Among a systematic literature review (refer to Appendix section), research has emphasised bamboo trusses in applications of greater structural dimensions (on the order of meters), in which whole culms or laminates manufactured from the plant are used. Some examples are illustrated in Fig. 1e. Spatial trusses on a smaller scale (tens of millimetres) and modularly made up of rods extracted from bamboo culms, as proposed in this manuscript, have not yet been addressed. The application of bamboo trusses as the core of a sandwich structure is even more scarce: the only research proposals employing sandwich design with distinct bamboo lattice cores were addressed in [46] and [47]; however, both manufacturing processes incorporate laminated bamboo—see Figs. 1e_5_ and e_6_, respectively. Another approach of a similar nature in the literature consists of a concrete sandwich beam, in which it is proposed to replace the interior truss, commonly made of steel, by a different one made of bamboo [48], but also in larger scale dimensions. Regarding bamboo trussed metastructures, there is no related research among the reported records, being only metamaterials bio-inspired by the bamboo morphology addressed so far [49, 50]. Therefore, it is noteworthy that the here-proposed sustainable metastructure cell for modular assembly is unprecedented in the indexed literature.

**Fig. 5:**
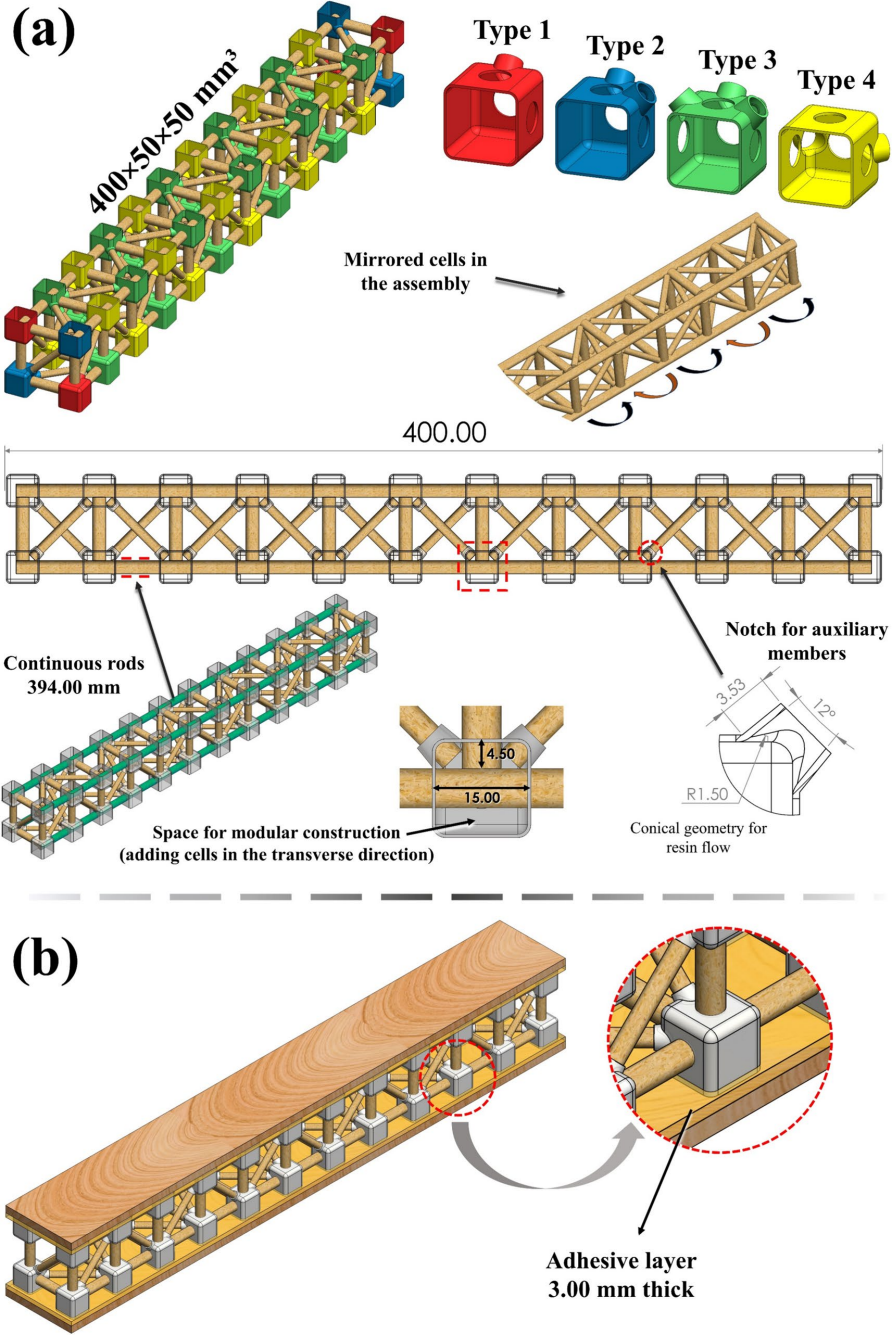
**a** Design details of the trussed beam and **b** assembly of the sandwich beam

**Fig. 6:**
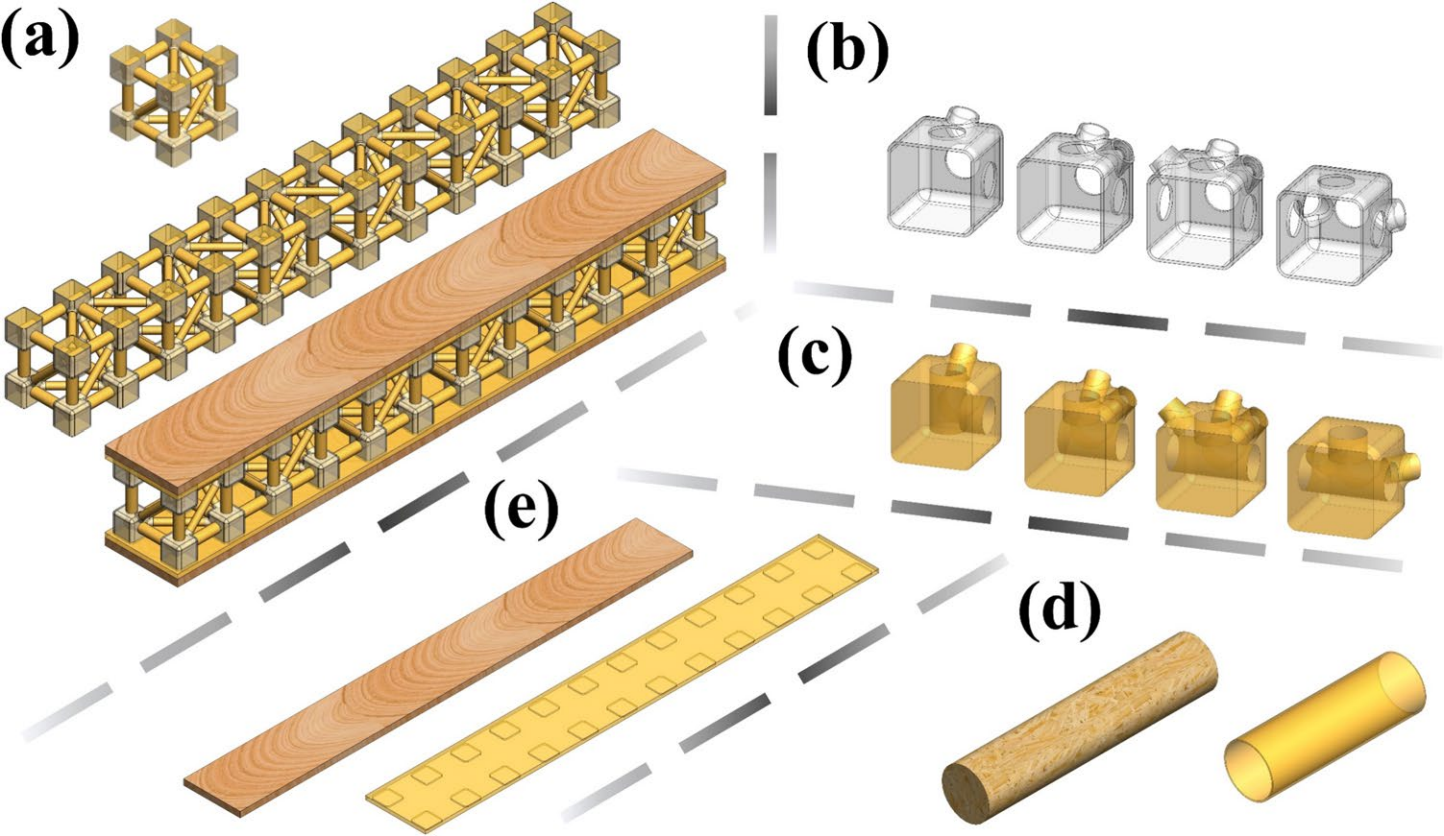
**a** 3D models and their constituent parts: **b** joint shells made of soybean-derived polymer, **c** joint filling formulated with castor oil-based polymer, **d** bamboo rod and its surface coating of castor oil polymer, and **e** balsa wood skin and adhesive layer consisting of castor oil polymer

### Specific research aims

Within the scope of the proposed research, each component of the metastructure trussed cell, composed of polymers and bamboo rods, undergoes individual testing to experimentally examine its physical, chemical, and mechanical properties. Prototypes of the trussed cell are then fabricated and subjected to compressive loading. The trussed beam is then modular constructed by linking these cells in series. The quasi-static response of the trussed beam is experimentally evaluated under four-point bending conditions. A four-point bending test is also conducted on a sandwich beam integrating the trussed beam with balsa wood skins. Experimental data obtained from these tests support the validation of finite element analysis (FEA) models that capture the elastic behaviour of these structures. These models are subsequently employed to simulate additional loading conditions, such as torsion and cantilever beams. A detailed outline of the specific objectives is presented in Fig. 3.

## Methodology

### Design

The 50 × 50 × 50 mm^3^ metastructure trussed cell is inspired by a 1:6 scale model of the metallic structure shown in Fig. 2. The mechanism of extraction of the bamboo rods used as members is properly addressed further in Sect. 2.2.1. The metastructure is assembled by twelve main members measuring 6 mm in diameter that occupy the position of the edges of the reference cube, jointly six auxiliary members measuring 4 mm in diameter that make up the diagonals—Fig. 4a. This design gives a triangular arrangement to each face of the cube. A perfect planar truss is then obtained on each face, to satisfy the classic algebraic relation [51] in which the number of members is equal to twice the number of joints minus three units ($5=2\times4-3$). The number of elements on each face of the cubic cell is therefore sufficient to avoid distortion of the geometric shape through external loads, without any redundancies that would imply an unnecessary increase in structural weight [52].

Through the isometric view in Fig. 4a, it is noteworthy that the modular design of the composite trussed cell allows its structure to rotate when compressed under loading perpendicular to the two horizontal faces of the cube, enabling it to function as a chiral metastructure capable of absorbing energy. This chiral behaviour is reminiscent of that observed in Kresling origami, a class of structures defined by a repeating triangular tessellation [53, 54], which may possess coupled axial compression and torsional deformation during folding. In the trussed cell, this behaviour is due to the positioning of auxiliary members making the diagonals on the four vertical faces of the cube in Fig. 4a, which are aligned with the expected direction of rotation. It can be seen that each extremity of eight of the main members has 4.5 mm of length inside each joint, as $\left(29.00-20.00\right)/2=4.50$; whilst for auxiliary members there is 5 mm, since $\left(37.00-27.00\right)/2=5.00$. There are four longer main members (41 mm), horizontally positioned in the isometric view in Fig. 4a, designed to transfer the load to the vertically arranged main members during compression of the cell. These four longer main members have 10.5 mm of each extremity embedded within the joints, as $\left(41.00-20.00\right)/2=10.50$. The joints are based on 15 × 15 × 15 mm^3^ elements and idealised in a two-phase structure: (i) 0.5 mm thick shells fabricated through a 3D printer using polymeric resin derived from soybean oil (cured by UV light), which act like cavities after connection with the members, being filled with (ii) bi-component polymeric resin based on castor oil (reaction cure), to promote the fixation of the truss elements. The microstructure of bamboo is naturally porous [34], allowing the fluid castor oil resin not only to fill the shells but also to be absorbed by the rods, providing a proper bond between the members within the joints. The detailed design of a shell element is depicted in Fig. 4b.

For the modular construction of the trussed beam with a length of 400 mm, four types of joints are used, differing from each other only by the relative position of the notches of the main and auxiliary members. Figure 5a shows the trimetric view of the trussed beam and the dimetric view of the shells of the four types of joints, identified by different colours. The joint previously detailed in Fig. 4b refers to Type 1, and the trussed cell in Fig. 4a is made by employing Types 1 and 2 joints. The series association of trussed cells that compound the trussed beam is performed along four continuous main rods, each with a length of 394 mm, as depicted in Fig. 5a. It is noteworthy that each face in the longitudinal direction of the beam has 45 members and 24 joints, so they are equally subject to the already mentioned algebraic relationship [51] for perfect planar trusses ($45=2\times24-3$). During assembly of the proposed arrangement, each cell is mirrored concerning the adjacent ones, providing a balance in the metastructure effect of each cell within the beam: if compressive efforts tend to rotate one cell clockwise, the adjacent cell tends to rotate counterclockwise.

The top view of the trussed beam in Fig. 5a evidences the cavities inside the shells of the joints, which have to be filled with the fluid polymeric resin derived from castor oil. The main members along the longitudinal direction of the beam are passersby and have 15 mm of their length within each joint. It is important to point out that, as a result of the continuity of these four rods, different cells have their main members in the longitudinal direction of the beam with a codependent force–displacement relationship. Nevertheless, the main members in both transverse directions of the beam remain independent and have 4.5 mm of length at each extremity inside the joints. In Fig. 5a, a notable highlight (dashed circle) is also given to the geometry for notching the auxiliary members: a trunk of a cone with an opening angle of 12° and a 1.5 mm radius fillet base, which must provide ideal adjustment during the flow of the fluid resin that should fill the shells and promote solid shape to the joints after curing. Finally, despite the modular construction by expanding the beam in the transverse direction not being part of the scope of the proposed research, it should be emphasised that the structure is designed by considering this possibility – see the free space within the joints in Fig. 5a.

The design of the sandwich beam, Fig. 5b, is based on previous published research [55, 56], within the scope of the RJS Method^1^for evaluation of the elastic behaviour of sandwich structures under bending, which is addressed with further detail in Sect. The RJS Method applied to the sandwich beam. The assumptions of the RJS Method are based on the classical mechanics of beams and cover concepts addressed by the ASTM standards C393 [57] and D7250 [58]. Maximising the flexural stiffness of sandwich structures involves the use of thicker skins (also denoted by faces or facesheets) or even those made of a more rigid material [55]. Concerning using thicker skins, it should be noted that the thickness of each face should not be greater than 10% of the core thickness, as reported in [56–58]. Otherwise, the transverse shear force under bending will be carried to a considerable extent by the skins, which goes against the purpose of the design of sandwich structures for load-bearing applications. Therefore, the novel sandwich beam is based on the association of the 50 mm thick trussed beam with 5 mm thick balsa wood skins, aiming to achieve the maximum stiffness without trespassing the limit of 10% of the core thickness when selecting the face thickness. Core-to-face bonding should be performed through the same castor oil polymer used to compound the joints of the trussed beam. The bi-component polyurethane derived from castor oil has been widely used to obtain polymeric compounds suitable for applications in materials and structural engineering, such as bonding skins and the core of sandwich panels [59–65].

As formerly reported, bamboo possesses a porous structure, capable of absorbing over 100% of its weight in water [34]. Therefore, all the proposed structures (cell, beam, and sandwich) are designed to undergo a final step during the fabrication, in which bamboo rods are manually coated with the polymer derived from castor oil through a hand-made brushing process, as properly described further in the manuscript (Sect. 2.2.3). The thin castor oil-based polymeric film of about 0.20 mm thick acts as a protective coating for the bamboo rods, safeguarding them against moisture and insect attack. Figure 6 presents highly accurate 3D models of the prototypes to be fabricated, which are designed in the SOLIDWORKS® CAD software (version SP3.1, 2022) [66]. These models provide a meticulous representation of each phase of the structural components: bamboo rods as members; joint shells and fills; bamboo rod coatings; and, for the sandwich beam, adhesive layers and balsa wood skins. These models serve as the basis for calculating key parameters such as the bulk volume of the structures. Furthermore, they are subsequently exported for modelling the finite element analysis to perform structural simulations.

### Fabrication

Section 2.2 covers the entire fabrication process, including the preparation of bamboo rods, 3D printing of joint shells, modular assembly of the structures, and finalisation with castor oil polymer. All phases of manufacturing are conducted in a controlled environment maintained at 23 °C and 55% relative humidity.

#### Bamboo rods

The bamboo rods are derived from the giant bamboo *Dendrocalamus asper*. The proximal section of the plant constitutes the primary region for obtaining natural composite rods, which have been extensively analysed in a prior study to statistically evaluate their physical and mechanical properties [34]. The proximal section, approximately the first 2.4 m from the initial usable culm above the soil, possesses the greatest wall thickness, making it particularly suited for the extraction of bamboo rods with transverse dimensions of 4 mm and 6 mm in diameter, which are integral to the fabrication of the metastructure trussed cells.

The proximal section contains six usable culms, with each inter-nodal segment of the plant considered as a distinct culm. For the here-proposed trussed design, rods are extracted from the fourth to sixth culms, as these exhibit superior mechanical properties, including higher elastic modulus and strength under tensile and compressive loading [34]. These culms also feature lengths between 400 and 500 mm, sufficient for producing all rods without the inclusion of bamboo nodes, encompassing the longer main members of 394 mm in length required for the trussed beam, as previously depicted in Fig. 5a. Bamboo nodes are deliberately excluded, as they are potential sites of structural weakness under certain loading conditions [67, 68].

The restriction on extracting continuous bamboo rods longer than 400 to 500 mm from the proximal section of the plant underscores the focus of this research on a 400 mm beam. Beams exceeding this mark require longer continuous bamboo rods, which are unavailable in the proximal section but can be sourced from further culms along the plant, which are longer than 500 mm [34]. Nevertheless, beyond the proximal section, the internal wall thickness of the bamboo begins to diminish, thereby constraining the maximum diameter of the rods that can be extracted [34]. An alternative could be to extract these longer rods from the proximal section of the bamboo by not discarding the bamboo nodes; however, as formerly pointed out, bamboo nodes are potential sites for structural weakness. The length of the trussed beam, in millimetres, follows the relation $35n+15$, depending on the number of cells ($n$). The beam can be constructed with an odd or even number of cells. For an odd number (as in the 400 mm trussed beam, which contains 11 cells), the beam’s mid-plane aligns with a cell’s mid-plane. For an even number of cells, the mid-plane of the beam coincides with truss joints.

Three mature bamboo plants (A, B, and C), approximately five years old, are selected, harvested, and conditioned for the extraction of prismatic cross-section rods through a band saw cutting mechanism, as comprehensively described in [34]. These rods depict a stable moisture content ranging between 11.51 and 11.66% at 23 °C and 55% relative humidity [34]. The process for obtaining cylindrical cross-section rods from the prismatic ones is explained in the next paragraph. To account for the biological variability and ensure statistical robustness of the mechanical performance, three independent sets of metastructure trussed cells are fabricated using rods from each plant (A, B, and C) and subjected to identical mechanical testing protocols. The dataset enabled the identification of the plant whose rod-derived structures displayed the most representative or typical mechanical response, which is then selected for the fabrication of the trussed and sandwich beams used in the subsequent experimental evaluations.

**Fig. 7:**
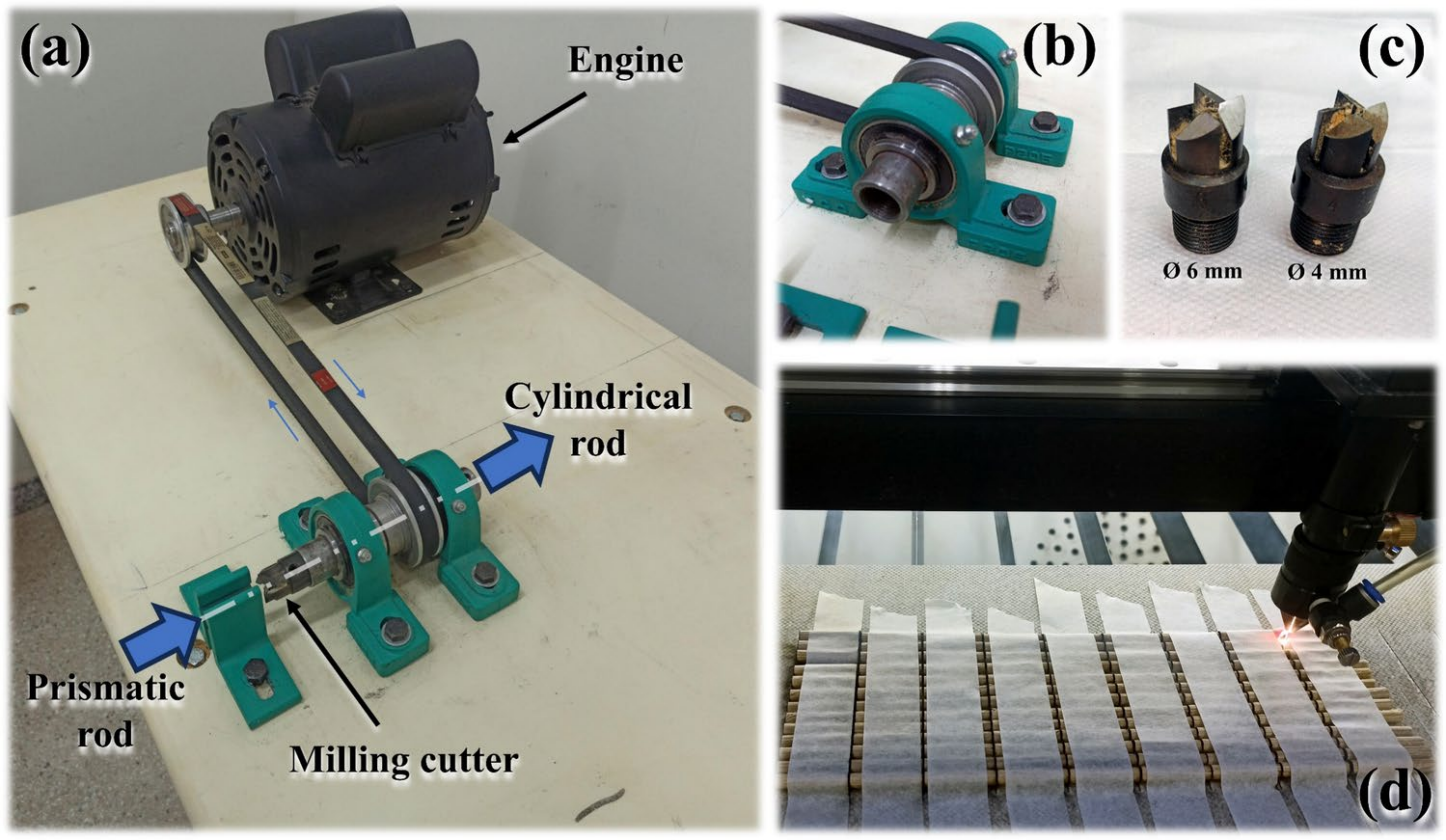
Process of obtaining cylindrical rods: **a** drawing system, **b** bearing apparatus, **c** milling tools, and **d** laser cutting

Bamboo rods with a square cross-section of 8 × 8 mm^2^ are processed into cylindrical rods with diameters of 4 mm and 6 mm (with a tolerance of ± 0.01 mm). A drawing system is used, driven by a 1 CV engine operating at 1745 rpm, comprising a bearing apparatus and milling cutters, as depicted in Fig. 7a–c. No chemical treatment is applied to the bamboo, aligning to achieve a sustainable design by utilising the natural composite rods extracted from the giant bamboo. To precisely ensure the length of each road to be used in the fabrication of the trussed structures, the rods are cut through a 150 W laser cutting machine (Jinan Robotec Machinery Co., Ltd.) configured with 80% power (120 W) and a speed of 20 mm/s—refer to Fig. 7d. For the main members of 394 mm, employed to fabricate the trussed beam as shown in Fig. 4a, the same laser machine is used at 12% power (18 W) and 100 mm/s speed without causing any damage to the rods, only to accurately mark the positions of the joints for the assembly of the structure.

#### 3D-printed joint shells

**Fig. 8:**
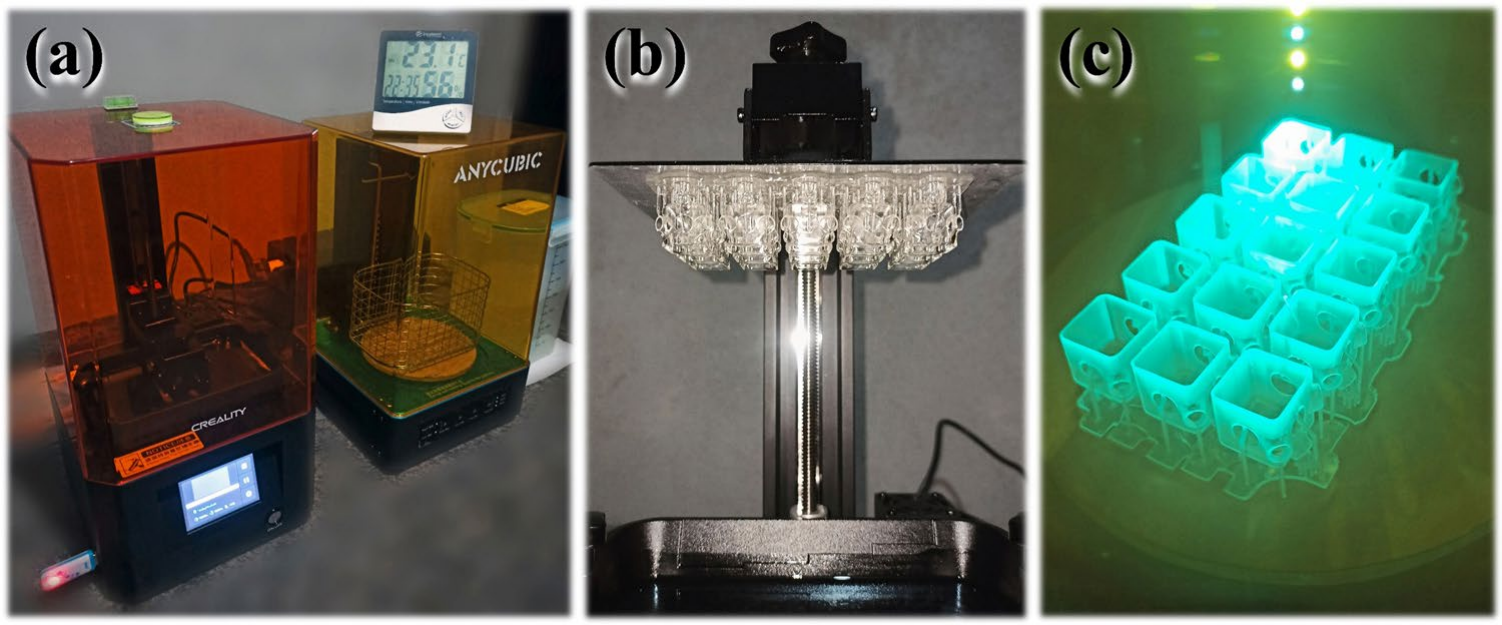
Overview of the 3D printing process: **a** 3D printer alongside the washing and curing machine, **b** 3D-printed joint shells, and **c** post-curing stage

A 3D resin printer using UV curing and an LCD screen operates by selectively curing liquid photopolymer resin into solid layers. The LCD screen acts as a mask, displaying the cross-sectional pattern of the object being printed. UV light passes through the transparent regions of the screen, curing the resin only where exposure occurs. This layer-by-layer process is repeated as the build platform incrementally moves, creating precise and highly detailed structures with excellent surface finish. The Anycubic® plant-based UV clear resin, derived from soybean oil, serves as the raw material for fabricating the shell components designed to connect the rods in the proposed trussed structures. The manufacturing process utilises a Creality® LD-002R 3D printer in conjunction with an Anycubic® Wash & Cure machine (50 W)—Fig. 8a.

The joint shells are fabricated by layers of 0.05 mm in thickness, the maximum permissible for the 3D printer employed. This increased layer thickness was selected to minimise printing time, thereby reducing energy consumption in alignment with sustainable design principles. The slicing of the shell components, previously modelled in 3D CAD, is performed using CHITUBOX® software [69], which generates automatic light supports. An exposure time of 10 s per layer is applied during the 3D printing process, as recommended by the resin manufacturer. The formerly detailed shell design, illustrated in Fig. 4b, incorporates tolerance variables for the holes intended to accommodate the main and auxiliary members. Following the extraction of the bamboo rods and the fabrication of prototype shells for testing, the established tolerances are defined to be + 0.03 mm for the main members and + 0.02 mm for the auxiliary members. Figure 8b depicts the 3D-printed joint shells. Subsequently, and in adherence to the resin manufacturer’s guidelines, the shells undergo a 2-min wash in isopropyl alcohol, followed by a 2-min post-curing process using UV light in the curing machine, as shown in Fig. 8c.

#### Assembly of trussed structures

**Fig. 9:**
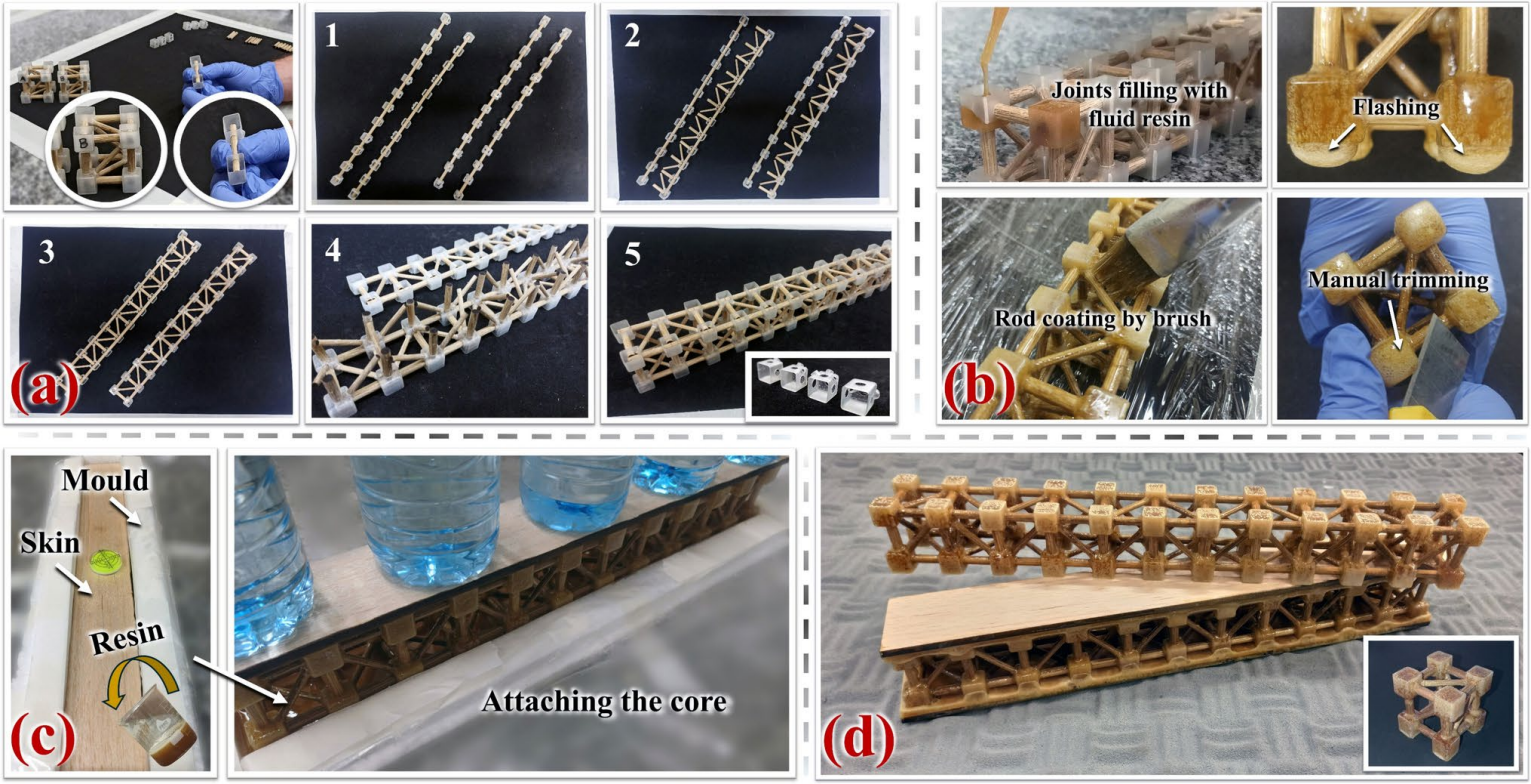
Fabrication sequence of the trussed structures: **a** manual assembly of the metastructure cell and trussed beam using bamboo rods and the soybean-based shell components, **b** application of castor oil-derived fluid resin for joint filling and rod coating, **c** moulding process for the production of the sandwich beam, and **d** fabricated prototypes

Figure 9a illustrates the manual assembly process of the metastructure trussed cell and trussed beam, utilising bamboo rods in conjunction with the four types of soybean-based 3D-printed shells. The assembly of the trussed beam is carried out in five distinct steps. Posteriorly, the castor oil-based polyurethane polymer (AGT 1315), supplied by Imperveg® (Brazil), serves as the bonding phase. This two-component adhesive comprises a pre-polymer and a polyol mixed at a 1:1.2 mass ratio. Figure 9b demonstrates the procedure for filling the cavities formed by the soybean-based shells via the manual casting of the castor oil-based resin. This step secures the truss elements in place, with the casting process performed sequentially: initially for the shells positioned in the same plane, followed by those in the opposite plane after a 24-h touch-dry interval.

The same castor oil-based resin is manually applied using a brush to form a protective coating on bamboo rods to improve their resistance to moisture and insect attack—Fig. 9b. Post-application measurements with a digital calliper indicate that the coating is 0.20 mm thick (± 0.02). It is important to highlight that the castor oil polymer is susceptible to bubble formation during the curing process [61]. Additionally, as per the resin manufacturer’s guidance, the presence of moisture can increase this effect, inducing an expansion of the polyurethane. Figure 9b reveals that the confined space within the shells, combined with the bamboo’s moisture content (ranging from 11.51 to 11.66%, as previously reported in Sect. 2.2.1), promotes the polyurethane expansion during curing. This results in the formation of flashing, which needs manual trimming post-touch-dry time for its removal.

Figure 9c depicts the fabrication process of the sandwich beam, which involves bonding balsa wood skins to the trussed beam using the castor oil-based polymer through a moulding technique. The 406 × 54 mm^2^ skins are cut from commercial 5.15 mm (± 0.02) thick sheets using the same laser cutting machine employed for the bamboo rods, but configured at 50% power (75 W) and a speed of 30 mm/s. The grain direction of balsa is aligned parallel to the longitudinal axis of the beam (i.e., along its length of 406 mm). Initially, the first skin is secured to the mould, and a layer of fluid resin is evenly applied to its surface. The trussed core is then placed on top of the resin layer, followed by the application of a compressive load of 1.28 MPa for 24 h. After this touch-dry curing period, the procedure is repeated for the second skin. The adhesive layer was initially designed to have a thickness of 1.50 mm, i.e., being in overlap with 10% of the joints’ dimension (15 mm). However, during fabrication, it was observed that the bubble formation and expansion characteristic of the castor oil polyurethane resulted in each adhesive layer with an average thickness of 3.09 mm (± 0.38), being ~ 1.23 mm between the skin and the core and ~ 1.86 mm in overlap with the joints of the truss. The thickness of the adhesive layers is measured through the ImageJ software (version 1.54k) [70]. The final thickness of the sandwich structure is 64.03 mm (± 0.41).

Figure 9d shows the fabricated prototypes of the trussed structures. A total of nine metastructure trussed cells are constructed, with an equal distribution of three cells fabricated from rods derived from each of the three sample bamboo plants (designated as A, B, and C), as previously outlined in Sect. 2.2. Additionally, three prototypes of each of the trussed beam and sandwich beam configurations are fabricated using rods sourced from bamboo plant B, which exhibited representative mean mechanical behaviour, as comprehensively detailed in subsequent sections.

According to the manufacturer, Imperveg®, the castor oil polymer system requires a curing period of 14 days (two weeks) to achieve complete polymerisation. However, the technical datasheet primarily pertains to its use in thin-film applications for waterproofing, such as the 0.2 mm coating here applied to the bamboo rods as members of the trussed structures. It is important to note that, in the proposed research, the castor oil polymer system is employed in more demanding configurations, including as a joint filler (15 × 15 × 15 mm^3^) and as a thick adhesive layer (3 mm) bonding the balsawood skins of the sandwich beam to its trussed core. These thicker applications may influence the curing kinetics, potentially extending the required curing time due to restricted air exposure and slower diffusion of reactive components (polyol and prepolymer). In real-world applications involving structural adhesives, it is critical to ensure full polymerisation to guarantee mechanical reliability and avoid premature failure due to incomplete curing. Therefore, to ensure that the polymer is fully cured, the prototypes of the here-proposed trussed structures are stored for 24 weeks prior to testing. This extended period serves as a conservative consideration equivalent to a safety factor of 12, intended to ensure complete polymerisation across all material thicknesses under ambient conditions and to avoid any mechanical inconsistencies during structural testing. The influence of castor oil polyurethane thickness on curing time and long-term mechanical stability is currently the focus of ongoing investigations within the research group responsible for the current manuscript, which aims to establish more precise curing protocols for varied geometries and application scales.

### Characterisation

Section 2.3 details the characterisation processes, from the raw materials to the trussed structures. All the tests are conducted in a controlled environment maintained at 23 °C and 55% relative humidity. Concerning the mechanical tests, the processing of force–displacement curves is performed using patented dedicated scripts of own authorship [71, 72]. The scripts’ algorithms are implemented in MATLAB® software (version R2022a) [73], and function similarly to the analysis software embedded in universal testing machines, enabling the extraction of key mechanical properties such as elasticity, yield, strength, resilience, and toughness. However, these scripts are tailored for batch processing of multiple samples, providing statistical outputs including mean and standard deviation, proper plots, and identifying the representative specimen (defined as the one whose target property is closest to the mean value of the sample set tested). Examples of such outputs can be found in [71, 72], and the scripts have been successfully applied in prior studies [12, 34, 60]. When applicable, statistical analyses of sample data (including comparative evaluations of means) are conducted using Minitab® software (version 20, 2021) [74].

#### Raw materials

The bamboo rods and polymers that compound the proposed metastructure trussed cell are characterised by employing chemical, physical, and mechanical tests.

##### Chemical analysis

To chemically characterise the raw materials used in the fabrication of the metastructure trussed cell, Fourier Transform Infrared Spectroscopy (FTIR) analysis is performed. The FTIR offers insights into the chemical compatibility, to assess a potential interfacial adhesion by identifying shared functional groups between the castor oil polymer (bonding phase) and the bamboo rods and soybean-based joint shells. FTIR spectra are obtained using a PerkinElmer® Spectrum 100 spectrometer. The scanning frequency range is set from 600 to 6000 cm^−1^, with a spectral resolution of 4 cm^−1^, and the results are obtained by averaging 32 scans.

##### Physical and mechanical analysis

The physical characterisation of the raw materials entails estimating the bulk density, calculated as the ratio of mass to volume. Mass is measured with a precision digital scale (0.0001 g), while volume is determined using a digital calliper (0.01 mm). Bamboo rod specimens, prepared for physical assessment, measure 30 mm in length and 6 mm in diameter; whilst both polymer materials are assessed using specimens of 15 × 15 × 15 mm^3^. These dimensions are compatible with the ones used in the proposed design of trussed structures. For the bamboo rods, thirty specimens are tested per plant (A, B, and C), evenly distributed among the 4th, 5th, and 6th culms. For both polymer types, fifteen specimens are examined, divided evenly across three replicates.

**Fig. 10:**
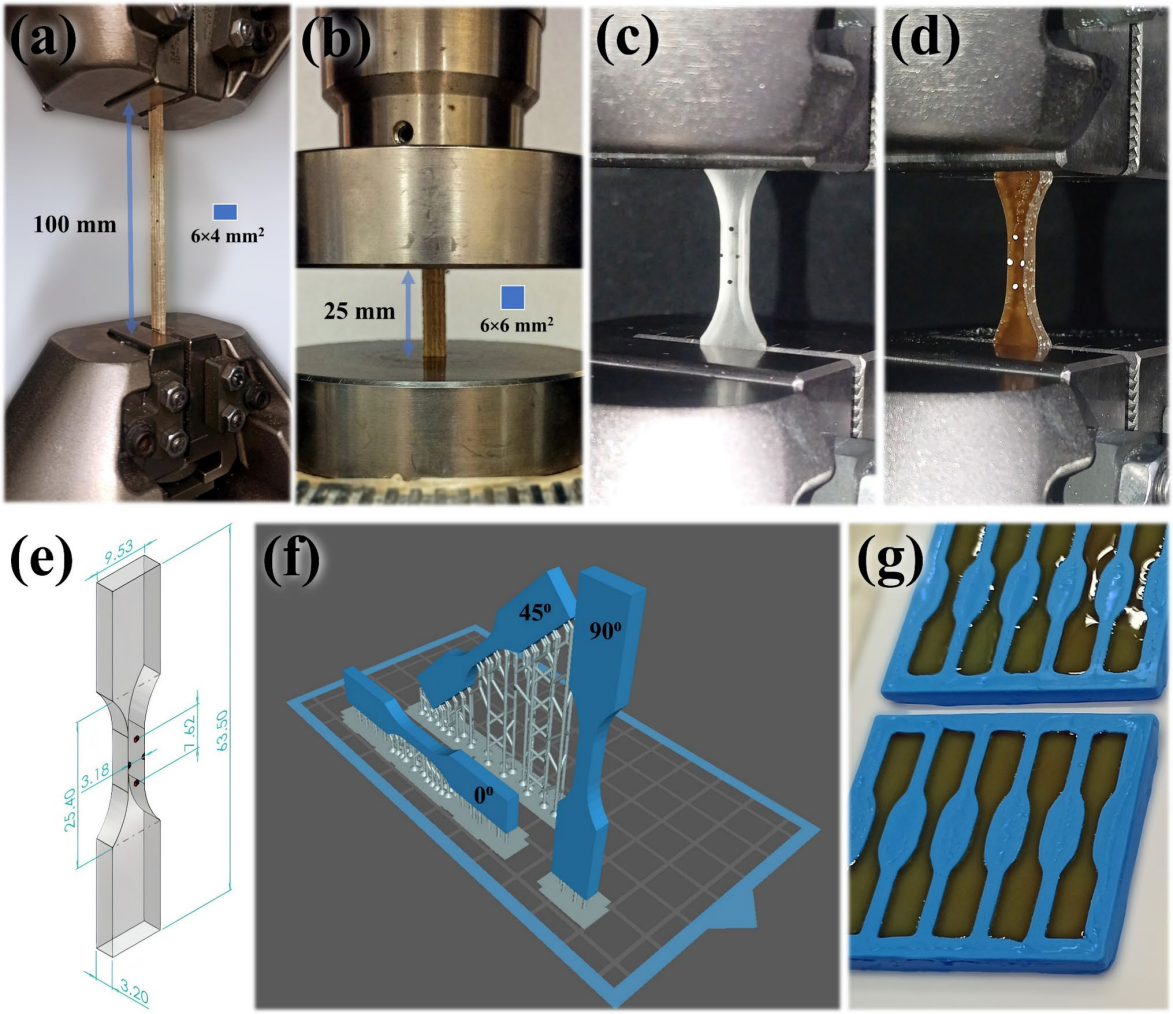
Insights on the mechanical tests of raw materials: **a** tensile test of the bamboo rod, **b** compression test of the bamboo rod, **c** tensile test of the soybean polymer, **d** tensile test of the castor oil polymer, **e** specimen dimensions for polymers testing, **f** three orientations for the tensile specimens of soybean polymer, and **g** moulding specimens of castor oil polymer

Bamboo rods are subjected to tensile and compressive testing as they constitute the members of the trussed structures, which are intrinsically exposed to one of these two types of loading. Furthermore, as a natural unidirectional fibre-reinforced composite, the bamboo rods exhibit varying properties under tension and compression. The rods are extracted and characterised following previous research [34]. Tensile specimens measure 200 mm in length, with a grip separation of 100 mm, and a cross-section of 6 × 4 mm^2^, as illustrated in Fig. 10a. Compressive specimens have a length of 25 mm and a cross-section dimension of 6 × 6 mm^2^, as depicted in Fig. 10b. The tensile and compressive testing protocols adhere to the ASTM D4761 standard [75], with test speeds of 4 mm/min and 1 mm/min, respectively (for a testing time of approximately 1 min, as specified by the standard). Both tests are carried out using a Shimadzu testing machine equipped with a 100 kN load cell. As well as applied for the physical assessment, both tests are performed for thirty specimens per plant (A, B, and C), equally distributed among the 4th to 6th culms. Properties of interest include elastic modulus, yield stress (at the limit of proportionality, defined by the classical 0.2% offset method^2^), ultimate stress, and Poisson ratio.

In contrast to the bamboo rods, both polymer materials (soybean- and castor oil-derived) are tested under tensile, using an Instron® testing machine equipped with a 1 kN load cell, at a speed of 1 mm/min – see Fig. 10c and d. The dimensions for testing the polymer materials follow the Type V test specimen proposed in the scope of the ASTM D638 standard [76]—Fig. 10e. The soybean-based polymer is fabricated via 3D printing as early described in the manuscript (refer to Sect. 2.2.2). However, as this is a layer-by-layer process, specimens are produced by three different orientations (0°, 45°, and 90°) relative to the loading direction to ensure the account for the possible influence of this parameter of fabrication—Fig. 10f. Five specimens are tested per orientation, totalling fifteen runs. Castor oil polymer specimens are produced through manual casting in silicone moulds, adhering to an identical curing duration (24 weeks) as employed for the trussed structures, with fifteen specimens evenly distributed into three replicates—Fig. 10g. The target properties for both polymer materials incorporate elastic modulus, ultimate stress, and Poisson ratio.

##### Supplementary mechanical tests

**Fig. 11:**
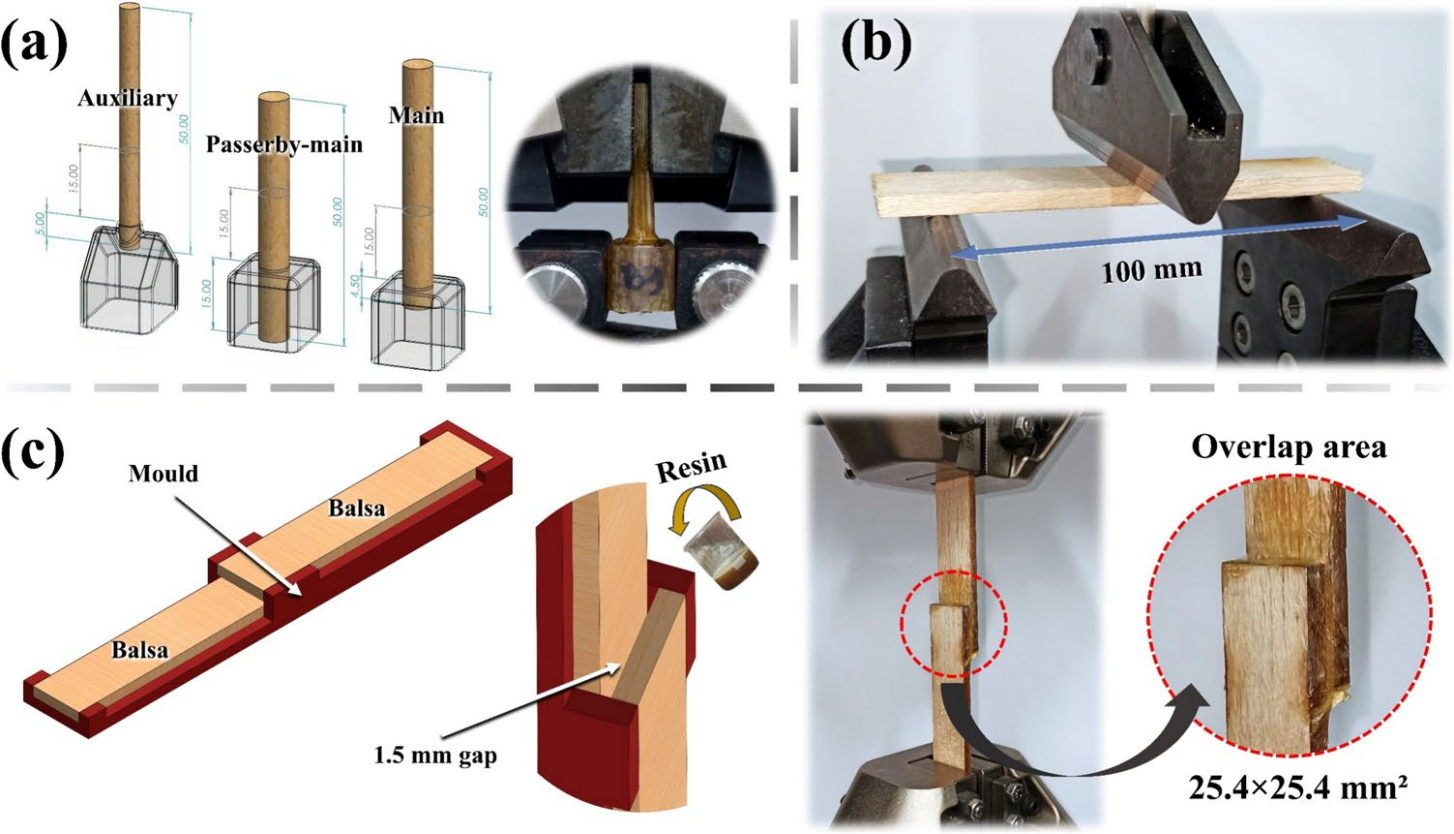
Supplementary mechanical tests: **a** illustration and testing of pullout specimens, **b** three-point bending of balsa wood sheet, and **c** preparation and testing of lap-shear specimens

To evaluate the mechanical performance of the bond between bamboo rods and the bi-phasic joints in the proposed structures, pullout tests are conducted—refer to Fig. 11a. Three configurations are employed to replicate the attachment of rods to the joints in the trussed beam: (i) an auxiliary rod with a diameter of 4 mm and an embedded length of 5 mm within the joint, (ii) a passerby-main rod with a diameter of 6 mm and an embedded length of 15 mm within the joint, and (iii) a main rod with a diameter of 6 mm and an embedded length of 4.5 mm within the joint. The specimens are fabricated via the same previously reported manual casting of the castor oil-based resin within the soybean shells with the attached rods—refer to Fig. 9b. These pullout tests are performed using an Instron® testing machine fitted with a 50 kN load cell, following the same 24-week curing period for the castor oil polymer, as performed for the trussed structures. The testing speeds are set at 2 mm/min for the auxiliary rod, 6 mm/min for the passerby-main rod, and 3 mm/min for the main rod. These speeds are defined to ensure that testing times remain between 1 and 2 min across all pullout conditions and specimens. Nine specimens are tested for each pullout configuration, equally divided into three groups (A, B, and C), which correspond to test specimens fabricated with rods extracted from the bamboo plants A, B, and C. The maximum load supported by the three configurations is the property of interest through the pullout tests.

The balsa wood used as the skins of the sandwich beam is characterised by its bulk density, which is determined through the mass-to-volume ratio of the six prepared skins – two for each of the three sandwich beam prototypes. A three-point bending test, also conducted following the ASTM D4761 standard [75], is then performed on five specimens with dimensions of 150 × 50 × 5 mm^3^, a support span of 100 mm, and a testing speed of 5.5 mm/min, on an Instron® testing machine equipped with a 1 kN load cell—Fig. 11b. This procedure serves to assess the elastic modulus along the grain direction of balsa and measure its flexural strength. With the bulk density and the elastic modulus along the grain direction determined, the elastic moduli, shear moduli, and Poisson ratios for the three principal directions of the laminae (given balsa’s inherent anisotropy) are estimated using the curves depicted within the consolidated multiscale model for elastic properties of balsa wood proposed by Malek and Gibson [77]. The curves represent the variation of elastic properties as a function of balsa wood density, enabling the association of experimental density values with estimated target elastic properties.

To assess the adhesive strength of the castor oil polymer to the balsa wood sheet, as it is employed in the sandwich beam, a lap-shear by tensile loading is performed in a Shimadzu testing machine with a 100 kN load cell. Figure 11c illustrates the process of fabricating the lap-shear specimens with a 25.4 × 25.4 mm^2^ overlap area, as well as the test conducted at 1.27 mm/min following the ASTM D3163 standard [78]. As with the other processes involving castor oil resin, the fabrication is carried out through manual casting, and the tests are performed following a 24-week curing period. The shear stress is the target property (calculated as the maximum load divided by the overlap area), by testing six specimens.

It is worth noting that the expansion of the castor oil polymer, both in filling the joint shells for the pullout samples and in the fabrication of the lap-shear specimens, occurs in the same manner as observed during the fabrication process of the trussed structures, requiring, therefore, the manual trimming process, as previously depicted in Fig. 9b. For the lap-shear specimens, plastic film and mould release wax are used to ensure that, during expansion, the castor oil polymer does not bond with any part of the balsa other than the designed overlap area.

#### Trussed structures

Both equivalent and bulk densities of the trussed metastructures are evaluated. The equivalent density is defined as the ratio of mass to the equivalent (homogenised) volume. The equivalent volume is determined as the product of the overall external dimensions of the structures (e.g., 50 × 50 × 50 mm^3^ for the trussed cells), effectively treating the structure as a homogenised continuum. In contrast, the bulk density is calculated based on the bulk volume, which corresponds to the material volume estimated through CAD software using the highly detailed 3D models of the prototypes described.

**Fig. 12:**
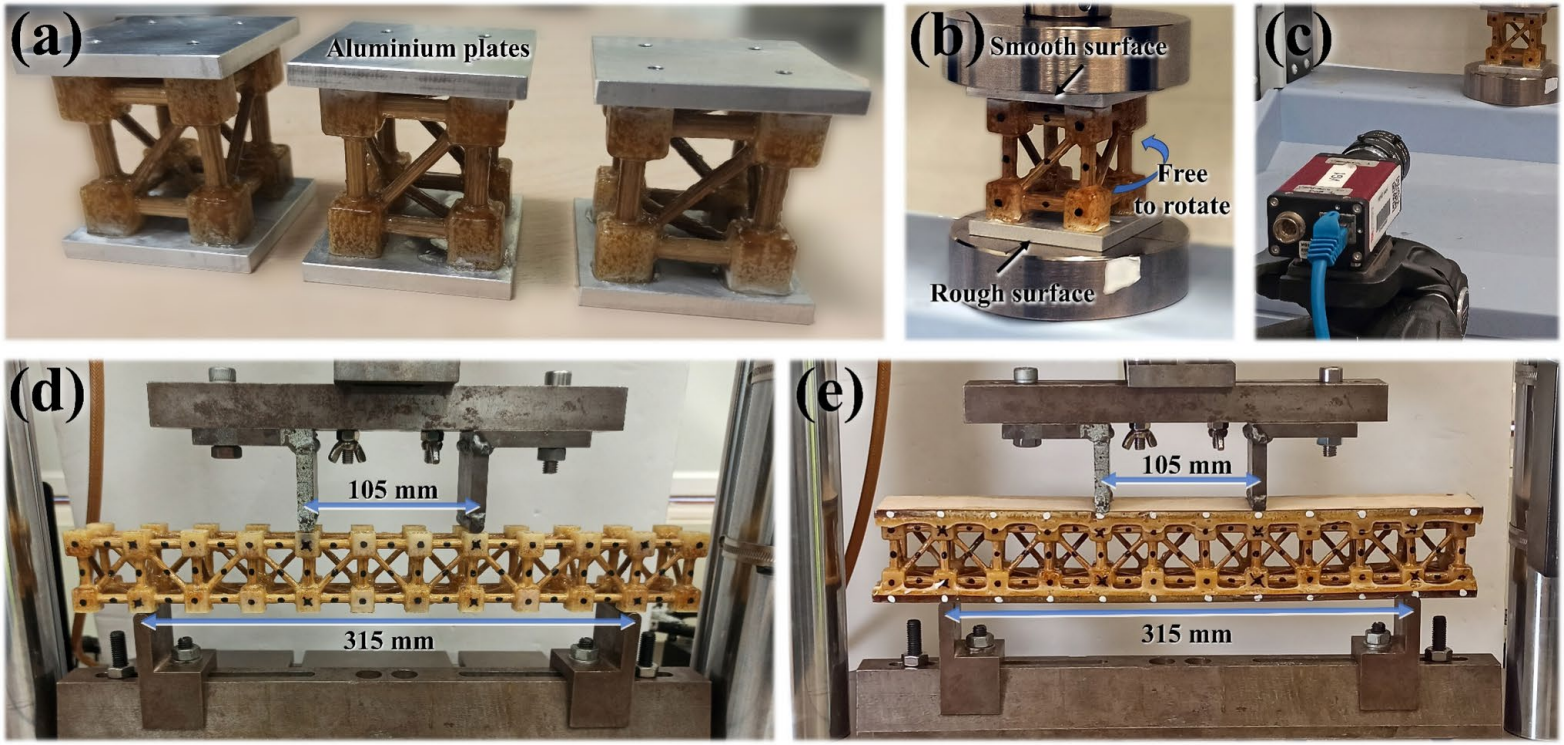
Insights on the mechanical characterisation of the trussed structures: **a** metastructure trussed cells attached to aluminium plates, **b** compression test of the trussed cell, **c** video gauge, **d** four-point bending of the trussed beam, and **e** four-point bending of the sandwich beam

For the compression testing of the metastructure trussed cells, aluminium plates (60 × 60 × 6 mm^3^) are adhered (using super glue) to both the top and bottom sections of the structure to ensure uniform load distribution. The rotation mechanism is facilitated by the bottom plate, which features a rough surface to establish stable contact, while the top plate has a smooth surface to permit free rotation under compressive loading—refer to Fig. 12a and b. The compression test is conducted using a Shimadzu testing machine equipped with a 10 kN load cell, with a crosshead speed of 1 mm/min (analogous to the compression of bamboo rods). To accurately capture the effect of cell rotation, with the bottom plate fixed and the top plate free to rotate, an Imetrum Video Gauge™ system is utilised to record displacement data. This is achieved by tracking black marker points placed on the trussed cells—see Fig. 12b and c. The Video Gauge™ system is similarly employed for precise data acquisition during the testing of trussed and sandwich beams under a four-point bending configuration. This setup incorporates a third-span loading arrangement, comprising a support span of 315 mm and a load span of 115 mm, as illustrated in Fig. 12d and e. The tests are performed at a speed rate of 2.5 mm/min (mean speed based on the tensile and compression tests of the bamboo rods) using an Instron® machine equipped with a 25 kN load cell.

##### Approach for the trussed cell

The compression behaviour of the metastructure trussed cell is initially evaluated through the force–displacement data, encompassing key parameters such as the slope of the linear portion of the curve, the maximum load, the maximum crosshead displacement (overall displacement of the structure), and the rotational degree of the metastructure calculated from the displacement of the marked points recorded through the Video Gauge™. Additionally, the stress–strain response is examined following the set of equations in (1). Stress ($\sigma$) is calculated by dividing the compressive force ($F$) by the cross-sectional area ($A$), while strain ($\varepsilon$) is determined using the crosshead displacement under compression ($\Delta$) and the length of the specimen ($L =$ 50 mm). In the process of normalising the force–displacement data to stress–strain, two distinct approaches are considered by varying the cross-sectional area ($A$). The first approach, termed “effective stress”, assumes the material area at the midsection of the structure under compression. This area is calculated using the 3D model in CAD software, by means of the intersection area of the trussed cell with the imaginary middle plane perpendicular to the load direction. The second approach, referred to as homogenised stress, considers the cross-sectional area of the trussed cell as a homogenised unit, i.e., defined as 50 × 50 mm^2^.

1 $$\left\{\begin{array}{l}\sigma =\frac{F}{A}\\ \varepsilon =100 \times \frac{\Delta}{L}\end{array}\right.$$

The stress–strain curves of the metastructure trussed cells under compression are used to evaluate the following properties: (i) the elastic modulus, determined from the slope of the linear region; (ii) yield stress and strain, obtained at the yield point (limit of proportionality), which is approximated using the 0.2% offset method; (iii) ultimate stress and strain, defined at the peak of the curves; and (iv) the moduli of resilience and toughness, which correspond to the areas under the stress–strain curves up to the yield and peak points, respectively. Area calculations are carried out using the trapezoidal rule in MATLAB® software [73].

##### Approach for the trussed and sandwich beams

The four-point bending performance of the trussed and sandwich beams is evaluated through the force–displacement data, encompassing the slope of the linear portion of the curve, the maximum load, and the maximum beam displacement (central deflection). The central deflection under bending is estimated from the marked points recorded across the Video Gauge™. Moreover, for the middle section of the beams (region of maximum deflection), the bending moment ($M$) and the mean stress–strain response (${\sigma }_{mean}$ vs. ${\varepsilon }_{mean}$) is assessed by following the set of equations in (2) – refer to [79]. The variable $F$ indicates the overall force applied during the bending test, whilst $S=315$ mm and $L=105$ mm account for the support and load span lengths, respectively. The thickness of the beams is represented by the variable $h$, while $I$ accounts for the area moment of inertia and $\Delta$ is the central deflection. The trussed and sandwich beams depict a variable moment of inertia for different cross-sections. Therefore, for estimating the mean stress–strain response, the homogenised moment of inertia is considered ($I=b\cdot {h}^{3}/12$), in which $b$ refers to the width of the beam. An effective stress approach, analogous to that employed for the trussed cell, could be implemented by considering the area moment of inertia at the midsection of the beam along the bending axis. However, unlike the cell (in which the midplane consistently represents the same geometry), the midsection moment of inertia of the beams varies with the number of cells along their length. As formerly reported in Sect. 2.2.1, for beams constructed with an even number of cells, the midsection coincides with the joints’ position; whereas for beams with an odd number of cells (as in the case of the here-proposed 400 mm length beams), it will exclusively contain rod members (the same midsection of a single cell). Once the area moment of inertia in the beam’s mid-plane presents this intrinsic dependence on the number of cells, only the homogenised approach is considered for the trussed and sandwich beams, as it provides a simplified and efficient representation of the structural behaviour across different configurations, which may be addressed in future studies.

2 $$\left\{\begin{array}{l}M=\frac{F\cdot (S-L)}{4}\\ {\sigma}_{mean}=\frac{M\cdot h}{2I}\\ {\varepsilon}_{mean}=\frac{12\cdot h\cdot \Delta}{3{S}^{2}-{\left(S-L\right)}^{2}}\end{array}\right.$$

As well as performed for the metastructure trussed cell, the following properties are estimated through the mean stress–strain curve of the trussed beams under bending: (i) the flexural modulus, as the slope of the linear region; (ii) yield stress and strain, at the yield point approximated by the 0.2% offset method; (iii) ultimate stress and strain, at the peak of the curves; and (iv) the moduli of resilience and toughness, corresponding to the areas under the stress–strain curves up to the yield and fracture points, respectively.

##### The RJS Method applied to the sandwich beam

The RJS Method [55, 56] is based on the homogenised concept, assuming both the sandwich panel (or beam) and its core as structures with a homogeneous moment of inertia. The theoretical flexural modulus (${E}_{s}$) of symmetric sandwich panels, characterised by skins of identical thickness and composition, can be determined using Eq. (3). This modulus is expressed as a function of the elastic moduli of the face skins (${E}_{f}$) and the core (${E}_{c}$) under bending, as well as the total panel thickness ($h$) and the core thickness ($c$). In the context of the current manuscript, ${E}_{c}$ is the homogenised flexural modulus of the trussed beam.

3 $${E}_{s}=\frac{{E}_{f}\cdot \left({h}^{3}-{c}^{3}\right)}{{h}^{3}}+\frac{{E}_{c}{\cdot c}^{3}}{{h}^{3}}$$

In a four-point bending, the experimental flexural modulus $\left({\overset\smile{E} }_{s}\right)$ of the panel can be measured from the elastic region of the force–displacement curve using Eq. (4) – refer to [56]. In this equation, $S$ denotes the support span length, $L$ is the load span length, $b$ is the width of the panel, and $m$ represents the slope of the linear (elastic) portion of the curve. The result from Eq. (4) is the same as measuring the flexural modulus through the slope of the linear portion of the mean stress–strain curve after normalising the force–displacement curve through the previously depicted set of equations in (2), by considering the homogenised approach ($I=b\cdot {h}^{3}/12$).

4 $${\overset\smile{E} }_{s}=m\cdot \frac{{2S}^{3}-3S\cdot {L}^{2}+{L}^{3}}{8b\cdot {h}^{3}}$$

According to the RJS Method, comparing the theoretical flexural modulus (${E}_{s}$) with the experimental value ${\overset\smile{E} }_{s}$ provides a means to evaluate the extent of shear deformations occurring in the core within the elastic regime. A lower experimental modulus relative to the theoretical value reflects greater shear deformations. The alignment of the experimental flexural modulus with its theoretical counterpart indicates a predominance of pure bending in the elastic regime. Achieving pure bending is facilitated by larger specimen dimensions, specifically a greater support span length. Conversely, for panels with identical face compositions, a higher core shear rigidity reduces the size requirements for achieving pure bending conditions. This analysis underscores the critical interplay between core shear rigidity, specimen dimensions, and the bending behaviour of sandwich panels, offering valuable insights into their mechanical performance under flexural loading.

When comparing experimental and theoretical flexural moduli ${\overset\smile{E} }_{s}$ and ${E}_{s}$, the RJS Method [56] introduces the variable “pure bending amount”, denoted as $\Pi$. The pure bending amount, ranging from 0 to 1, is quantified as the ratio of the experimental flexural modulus to the theoretical flexural modulus, as expressed in Eq. (5). This metric provides a direct measure of the extent of pure bending, with higher values indicating reduced shear deformation and greater alignment with ideal bending conditions. Additionally, the pure bending amount can be expressed as a percentage by multiplying Eq. (5) by 100, offering a more intuitive interpretation in terms of percentage alignment with pure bending behaviour.

5 $$\Pi=\frac{{\overset\smile E}_s}{E_s}$$

The RJS Method [56] incorporates the estimation of the support span length ($S$) required to promote a certain amount of pure bending ($\Pi$), as described in (6). In the RJS approach, it is depicted that $\Pi =0.95$, i.e., 95% of pure bending, is enough to assume the shear deformations as negligible. Beyond this threshold, achieving further increments in the pure bending amount necessitates disproportionately large increases in the support span, yielding only marginal improvements in the pure bending amount. In (6), $r$ is the ratio between the load span and the support span ($L/S$). The variables $D$ and $U$ denote the flexural and the shear stiffnesses of the panel, respectively, both properties calculated using Eqs. (7) and (8), based on the ASTM D7250 standard [58], in which ${G}_{c}$​ corresponds to the core shear modulus. Only the first term of Eq. (7) pertains to ASTM, while the second term (dependent on ${E}_{c}$) is also attributed to the RJS Method, as it accounts for the contribution of core rigidity.

6 $$\left\{\begin{array}{c}S=4.90\Phi \sqrt{\frac{D}{U}} \\ \Phi =\sqrt{\frac{\Pi }{\left(\Pi -1\right)\cdot \left[{\left(r-1\right)}^{2}-3\right]}}\end{array}\right.$$

7 $$D={E}_{f}\cdot \frac{b\cdot \left({h}^{3}-{c}^{3}\right)}{12}+{E}_{c}\cdot \frac{{b\cdot c}^{3}}{12}$$

8 $$U=\frac{{G}_{c}\cdot {\left(\frac{h+c}{2}\right)}^{2}\cdot b}{c}$$

The core shear modulus can be experimentally measured by applying Eq. (9) to determine the shear stiffness ($U$) and then estimating ${G}_{c}$ by Eq. (8) – refer to [58]. The variables ${F}_{i}$ and ${\Delta }_{i}$ represent the force and displacement values, respectively, for each of the $n$ data points selected within the linear segment of the force–displacement curve. To ensure accuracy, a minimum of ten data points should be utilised. Equation (9) is most effectively applied to experimental loading configurations with less than 50% of pure bending ($\Pi <0.5$), i.e., a bending test with the predominance of shear deformations in the elastic regime [56].

9 $$U=\frac{\sum_{i=1}^{n}\frac{{F}_{i}\cdot (S-L)}{4\left[{\Delta }_{i}- \frac{{F}_{i}\cdot (2{S}^{3}-3S\cdot {L}^{2}+{L}^{3})}{96D}\right]}}{n}$$

Moreover, two innovative factors are presented within the scope of the RJS Method, which are: (i) the $\overline{\text{RJS} }$ factor for core rigidity relevance [55], and (ii) the ${\text{RJS}}^{*}$ factor for shear rigidity relevance [56]. The $\overline{\text{RJS} }$ factor, Eq. (10), represents the percentage contribution of the core to the flexural modulus of the sandwich structure under pure bending, relative to the contribution of the skin materials. The ${\text{RJS}}^{*}$ factor, Eq. (11), is a dimensionless quantity that compares the shear rigidity to the flexural rigidity of the sandwich beam. The higher the ${\text{RJS}}^{*}$ factor, the smaller the required increment in the support span to achieve 95% pure bending.

10 $$\overline{\text{RJS}}\left({\%}\right)=100\left[\frac{{E}_{c}}{{E}_{f}}\cdot \frac{{c}^{3}}{\left({\text{h}}^{3}-{c}^{3}\right)}\right]$$

11 $${\text{RJS}}^{*}={h}^{2}\cdot \frac{U}{D}$$

Finally, the RJS Method [56] classify the sandwich beams into three categories based on the bending behaviour, following the ratio between the support span length under bending ($S$) and the panel thickness ($h$). If $S/h<19$ and at least 95% of pure bending occurs, then the sandwich beam is classified as one with a very high rigidity core; if the loading configuration requires $19\le S/h\le 21$ to achieve at least 95% of pure bending, then the sandwich beam is classified as one with a non-negligible rigidity core; and, if $S/h>21$ is demanded to reach 95% of pure bending, then the panel has a negligible rigidity core. This pure bending behaviour, depending on the $S/h$ ratio, can be estimated through the ${\text{RJS}}^{*}$ factor and the loading configuration, specifically the ratio between the load and support span lengths – the $r$ variable depicted in (6). In the most critical case (three-point bending, $r=0$), higher ${\text{RJS}}^{*}$ values are required to achieve pure bending. As $r$ increases (four-point bending, with a greater load span length), shear effects are mitigated, allowing pure bending behaviour to be attained in panels with a lower ${\text{RJS}}^{*}$ factor. However, in general, covering all the cases for a sandwich beam [56], a negligible rigidity core is classified when ${\text{RJS}}^{*}<0.38$, whereas a very high rigidity core is defined by ${\text{RJS}}^{*}>0.63$.

The RJS Method has been successfully applied to assess the elastic properties of sandwich structures [59–63]. Therefore, all the concepts presented here concerning the RJS Method are systematically applied to the proposed sandwich beam, enabling a comprehensive analysis of its mechanical behaviour within the elastic regime, and ensuring that both theoretical and experimental frameworks are effectively integrated to evaluate its performance under bending.

### Finite element analysis

FEA models are proposed to capture the elastic behaviour of the trussed structures through the ABAQUS™ CAE software (version 2017) [80]. The assembled structures are the ones early designed in the SOLIDWORKS® [66], which are exported in the *Parasolid* format to ABAQUS. The models are based on the elastic properties of each raw material employed in the fabrication process (polymers, bamboo rods, and balsa wood), which are obtained from the experimental tests.

**Fig. 13:**
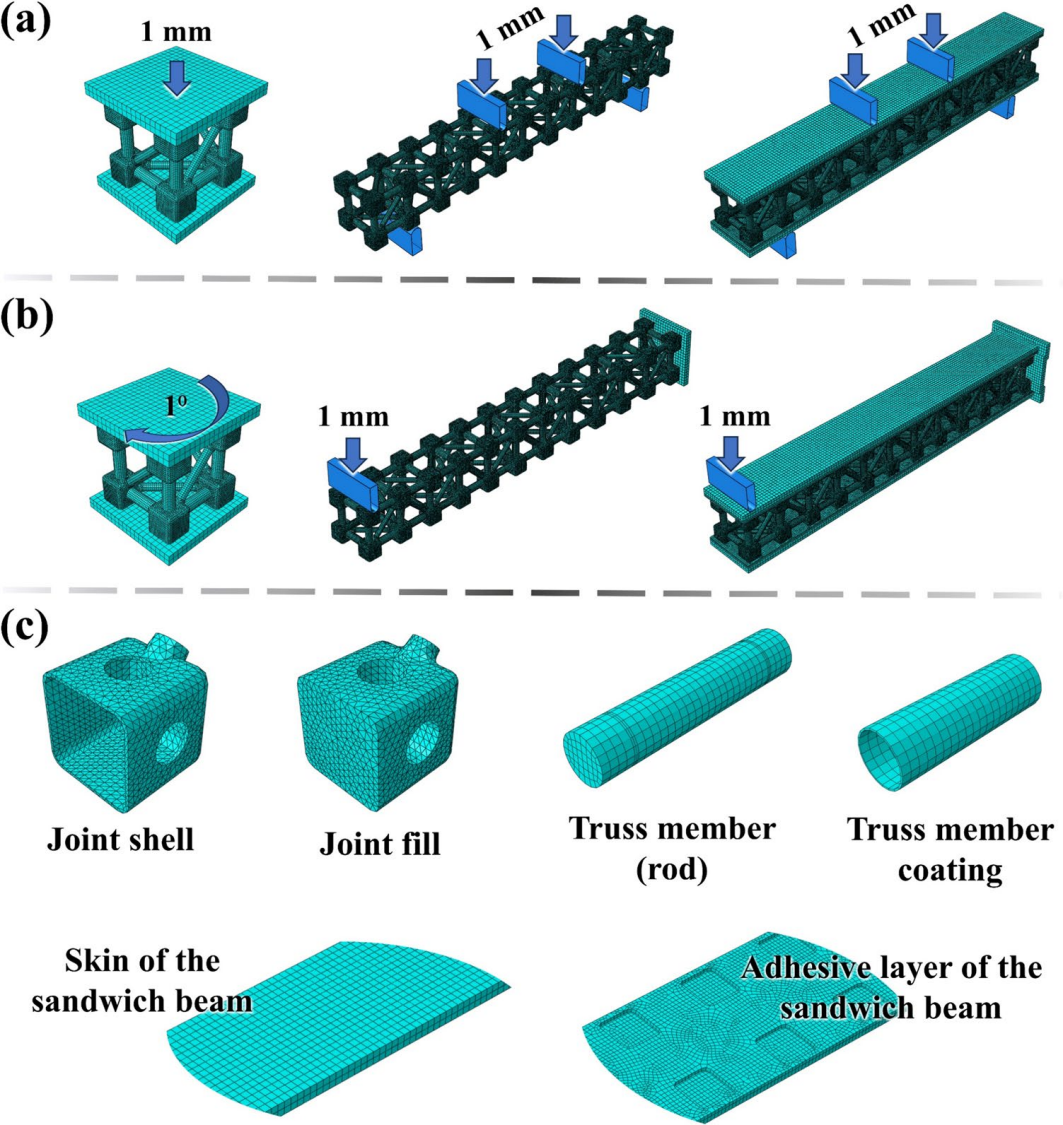
Insights on FEA analysis: **a** models for validation through the experimental data, **b** additional loading conditions evaluated through the numerical simulation, and **c** mesh of the parts used in the model assembly

The overall force–displacement response is validated through the experimental tests of the trussed structures, encompassing: (i) compression of the metastructure cell, (ii) four-point bending of the trussed beam, and (iii) four-point bending of the sandwich beam—refer to Fig. 13a. Once these FEA models are validated, they are employed to simulate additional loading conditions, such as torsion of the metastructure trussed cell, and bending of the trussed and sandwich beams through the cantilever configuration—Fig. 13b. The FEA analysis provides a meticulous representation of each part of the trussed structures, offering a detailed breakdown of its components: joint shells and fills; truss members (rods); members’ coating; and the adhesive layers and balsa wood skins applied to the sandwich configuration—Fig. 13c.

Table 1 summarises the input properties of each part to be included in the FEA, all of which are modelled as solid and homogeneous sections. Except for balsa wood, all materials are assumed to be isotropic and characterised by their density, elastic modulus, and Poisson ratio. In the here-proposed approach, the bamboo rods, despite being a natural composite material comprising unidirectional fibres, produced accurate results when modelled as isotropic. This simplification is justified by its application in trussed structures, where stresses predominantly act longitudinally (in tension or compression). A key adaptation is made to account for the significant variation in the elastic modulus of bamboo rods under tensile and compressive loading [34]. In the compression-dominated deformation of the metastructure trussed cells under compression and torsion, the elastic modulus of bamboo is set to its compressive value. Conversely, in the scenarios involving bending (trussed and sandwich beams), the tensile elastic modulus is considered. This distinction ensures the models accurately reflect the mechanical behaviour of the bamboo rods under different loading conditions. The balsa wood is modelled as an orthotropic material, characterised by its density and nine engineering constants. For the compression and torsion of the trussed cell, the support plates are defined as shell parts created from discrete rigid solids; and the fixtures for the four-point bending are analytical rigid extruded shells. The four-point bending fixtures are cylindrical in the surface of contact, with an 8 mm diameter for the loading fixtures and 12 mm for the support fixtures, following the experimental setup.

**Table 1:** Property details for each part of the FEA models

Part	Section type	Material	Behaviour	Properties
Joint shells	Solid, homogeneous	Soybean polymer	Isotropic	Density, Young’s modulus, and Poisson ratio
Joint fills		Castor oil polymer		
Truss members (rods)		Bamboo		
Truss member coatings		Castor oil polymer		
Skins of the sandwich beam		Balsa wood	Orthotropic*	Density, E1, E2, E3, Nu12, Nu13, Nu23, G12, G13, and G23
Adhesive layers of the sandwich beam		Castor oil polymer	Isotropic	Density, Young’s modulus, and Poisson ratio

^*^Ei accounts for the elastic modulus, Nuij is the Poisson ratio, and Gij is the shear modulus

The formation of bubbles during the curing process of the castor oil polymer when it is applied as the joint fills or the adhesive layers of the sandwich beams, as previously reported in Sect. 2.2.3, implies the need to consider, in the FEA models, the correction for the elastic modulus of the castor oil measured in the tensile tests referred in Sect. 2.3.1. By comparing the mass of the fabricated structures (measured step by step during the process of assembly, following Sect. 2.2.3) with the mass estimated through the 3D CAD models, it is observed that: (i) the castor oil polymer applied as the coating of the bamboo rods has the same density of the reference castor oil polymer (the specimens prepared for evaluation of density and tensile properties); (ii) the castor oil polymer as the fills of the joints has about 78% of the density compared to the reference; and (iii) the castor oil polymer as the adhesive layers of the sandwich beams has nearly 63% of the density of the reference castor oil.

For estimation of the proper elastic modulus to be considered in the FEA models in front of the castor oil polymer density reduction in the joint fills and adhesive layers, Eq. (12), from Gibson & Ashby [81], is applied. The elastic modulus of the porous material (${E}^{*}$) can be estimated through its density (${\rho }^{*}$), by knowing the elastic modulus and density of the reference solid (${E}_{r}$ and ${\rho }_{r}$). According to Gibson & Ashby [81], the constant $C$ is a factor for correction through experimental results, and it is usually equal to one unity ($C=1$). Nevertheless, lower values may be necessary if the material has a high irregularity constituted by large voids, which highly influence the overall mechanical performance under certain loading conditions. Several tests comparing the experimental and numerical results of the proposed trussed structures depicted that $C=1$ fits the correction for the castor oil filling the joints in the four-point bending of the trussed beams. In this setup, the loading is just transferred to the fills through the four joints in contact with the loading fixtures, which is then transferred to the bamboo rods; and the other joints and fills act only as connectors between rods. Concerning the compression of the trussed cells, all the joints and fills are directly and highly submitted to stresses, which are then transferred to the rods. The same occurs to the adhesive layers and the joint fills in the sandwich beam, those which directly receive the load transfer from the skins and transmit it to the rods. Therefore, for castor oil filling the joints in the trussed cell, and for the castor oil making the adhesive system to the skins and the joint fills of the sandwich beam, the value $C=0.1$ is the one depicted to be proper for the FEA analysis matching experimental results. It is important to note that Eq. (12) is intended for cellular materials. Given the bubble-like structure, the castor oil polymer studied here behaves more like a solid with voids. Even so, using the equation is reasonable because the constant $C$ values have been validated through both numerical and experimental results. Moreover, consistent behaviour is noted, as $C=0.1$ is observed exactly in the two different structures (cell and sandwich beam) in which the castor oil polymer undergoes higher strain rates, while $C=1$ applies to lower strain rates (trussed beam).

12 $$\frac{{E}^{*}}{{E}_{r}}=C\cdot {\left(\frac{{\rho }^{*}}{{\rho }_{r}}\right)}^{2}$$

Table 2 presents the mesh details for each part encompassed in the FEA, including the mesh size and total number of elements in each model. A conventional approach of mesh refinement and convergence analysis is not employed. Instead, the fills (the most geometrically complex components) are used as the reference for mesh design. The mesh size for the fills is reduced incrementally until the proportion of distorted elements is below 3% of the total elements in the part, ensuring convergence stability without excessively increasing the computational cost. This approach ensures a balance between accuracy and computational efficiency, which is particularly critical for simulations involving complex geometries like the fills. Based on this criterion, the fills receive a mesh size of 1 mm, and this pattern is applied to all other parts except for the adhesive layer (1.5 mm mesh) and the faces of the sandwich beams (3 mm mesh). It is noteworthy that the number of elements in the sandwich beam is lower than in the trussed beam. Although the sandwich beam includes additional components (skins and adhesives), this reduction is likely due to node merging or mesh simplification at shared interfaces or overlapping regions, particularly at the tie regions of the adhesives to the joint fills and shells.

**Table 2:** Mesh details for each part of the FEA models and its number of elements

Part	Mesh element type*	Mesh element family	Mesh size
Joint shells	C3D4	3D stress	1 mm
Joint fills			
Truss members (rods)	C3D8R		
Truss member coatings	SC8R	Continuum shell	
Skins of the sandwich beam	C3D8R	3D stress	3 mm
Adhesive layers of the sandwich beam			1.5 mm
FEA model		Number of elements	
Trussed cell		244,700	
Trussed beam		1,508,720	
Sandwich beam		1,342,452	

^*^C3D4: 4-node linear tetrahedron. C3D8R: 8-node linear brick, reduced integration, hourglass control. SC8R: 8-node quadrilateral in-plane general-purpose continuum shell, reduced integration with hourglass control, finite membrane strains. Tetrahedral elements are employed in parts of complex geometry, such as joint shells and fills, where meshing constraints rendered hexahedral meshing impractical or resulted in poor-quality elements. Conversely, in components with simpler geometries, structured hexahedral elements are utilised to enhance numerical accuracy and computational efficiency

Table 3 depicts insights into the modelling of the interaction between parts which compound the FEA models. As the focus lies in the elastic analysis (small deformations), a perfect bonding between phases is assumed through the constraint tie interaction. For the contacts between fixtures and structures in the four-point bending models, it is considered a penalty tangential behaviour with a friction coefficient of 0.2, and “Hard” contact normal behaviour. The discretisation method for the tie is “analysis default”, and for the fixtures and plates it is “node to surface”. Table 3 also points out the master and slave surfaces for each interaction pair. In ABAQUS™ [80] contact analysis, the master surface refers to the primary surface that controls the contact interaction, while the slave surface is the secondary surface that responds to the master surface’s movements. Typically, the slave surface should have a higher or the same degree of mesh refinement as the master surface.

**Table 3:** Interaction properties of the FEA models

FEA model	Interaction pair	Type of interaction	Master surface	Slave surface
All	Joint shells to fills	Constraint: tie	Fills	Shells
	Joint fills to rods		Rods	Fills
	Rods to coatings		Rods	Coatings
Trussed cell	Plates to shells		Plates	Shells
	Plates to fills		Plates	Fills
Trussed beam	Fixtures to shells	Contact*	Fixtures	Shells
	Fixtures to fills		Fixtures	Fills
Sandwich beam	Adhesives to shells	Constraint: tie	Adhesives	Shells
	Adhesives to fills		Adhesives	Fills
	Skins to adhesives		Skins	Adhesives
	Fixtures to skins	Contact*	Fixtures	Skins

^*^Penalty tangential behaviour with a friction coefficient of 0.2, and “Hard” contact normal behaviour

Finally, for all the proposed FEA models, the “Static, General” step (implicit analysis) is configured with automatic stabilisation, employing a specific dissipated energy fraction of 0.0002, a maximum ratio to strain energy of 0.05, and initial and minimum increment sizes of 0.01 and 1E-007, respectively. The reaction force is recorded for each increment up to a displacement of 1 mm for compression and bending models, which is within the elastic portion of the experimental curves as properly depicted further in the manuscript (Sects. 3.2.1 and 3.3.1). In the cantilever beam simulations, the load is applied at the central region of the terminal joint opposite the fixed support, corresponding to a position of 395 mm along the beam’s length. For torsion of the trussed cell, the reaction moment is recorded up to a twist of 1° (0.0175 rad). The torsion is applied in the opposite direction to the rotational tendency of the structure when compressed.

## Results and discussions

### Properties of raw materials

#### FTIR spectra

**Fig. 14:**
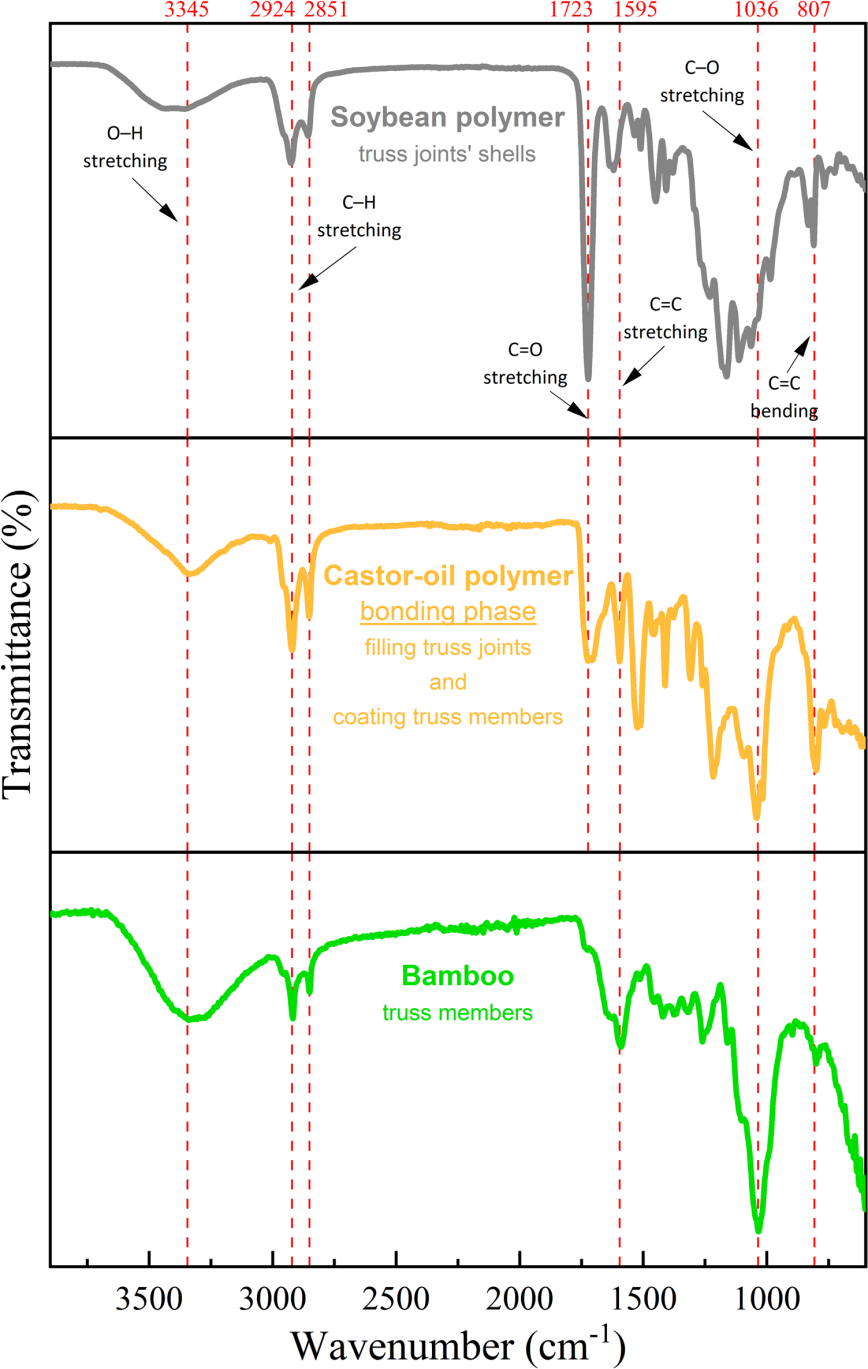
FTIR spectra for the raw materials compounding the metastructure trussed cell

Figure 14 reveals important insights into the FTIR spectra results, pointing out evidence of the chemical compatibility and potential adhesion mechanisms within the three-phase metastructure comprising bamboo rods, soybean polymer, and castor oil polymer. The shared transmittance peaks among the raw materials (particularly O–H, C–H, and C–O) suggest the presence of hydroxyl, alkyl, and ether functional groups, which may facilitate hydrogen bonding and intermolecular interactions across the interfaces. The C = C stretching is more pronounced in bamboo, which may indicate residual unsaturation in its lignocellulosic matrix, potentially offering sites for covalent bonding with reactive species in the castor oil polymer. Notably, the shared C = O stretching between the soybean and castor oil polymers underscores the potential for ester linkages or other carbonyl interactions that may enhance adhesion at their interface. Overall, the similar functional distribution of polar groups like O–H and C = O suggests the potential for strong interfacial bonding, improving the cohesive integrity of the composite structure.

#### Physical and mechanical properties

Table 4 summarises the physical and mechanical properties of the natural composite rods extracted from bamboo. The bulk density ranges from nearly 0.50 to 0.69 g/cm^3^. Under tensile loading, the elastic modulus varies between 7.84 and 10.74 GPa, the yield strength lies between 61.7 and 82.6 MPa, and the ultimate tensile strength spans from 122 to 177 MPa. Regarding compressive properties, the elastic modulus ranges from 2.11 to 3.55 GPa, the yield strength lies between 29.3 and 48.3 MPa, and the ultimate compressive strength varies from 32.0 to 51.7 MPa. Plant B exhibits mechanical behaviour representative of the overall mean response. In the rod configuration, characterised by a small transverse diameter relative to the rod length, transverse deformation is shown to be negligible in comparison to longitudinal deformation, resulting in an effective Poisson ratio of zero.

**Table 4:** Physical and mechanical properties of the bamboo rods

Bamboo plant	Bulk density (g/cm^3^)	Rod loading configuration	Elastic modulus (GPa)	Yield stress (MPa)	Ultimate stress (MPa)	Poisson ratio
A	0.502 ± 0.031	Tensile	7.84 ± 1.11	61.7 ± 8.5	122 ± 15	0
		Compression	2.11 ± 0.78	29.3 ± 4.6	32.0 ± 5.0	
B	0.646 ± 0.082	Tensile	9.41 ± 1.53	79.6 ± 14.2	144 ± 28	
		Compression	3.39 ± 1.10	47.2 ± 11.4	51.1 ± 10.2	
C	0.690 ± 0.041	Tensile	10.74 ± 1.16	82.6 ± 8.3	177 ± 17	
		Compression	3.55 ± 1.27	48.3 ± 7.3	51.7 ± 6.5	

Table 5 presents the physical and mechanical properties of soybean and castor oil-based polymers. The soybean polymer exhibits a bulk density of about 1.19 g/cm^3^, an elastic modulus of 1.20 GPa, an ultimate strength of 25 MPa, and a Poisson ratio of 0.33. The castor oil polymer depicts a bulk density of nearly 1.03 g/cm^3^, an elastic modulus of 825 MPa, an ultimate strength of 20.5 MPa, and a Poisson ratio of 0.45. Table 6 shows the corrected values for the castor oil properties to be considered in the FEA models, following the formerly depicted methodology in Sect. 2.4, based on Gibson & Ashby [81].

**Table 5:** Physical and mechanical properties of soybean and castor oil polymers

Polymer material	Bulk density (g/cm^3^)	Elastic modulus (GPa)	Ultimate stress (MPa)	Poisson ratio
Soybean	1.187 ± 0.008	1.20 ± 0.22	25.0 ± 6.3 *	0.33 ± 0.05
Castor oil	1.032 ± 0.008	0.825 ± 0.095	20.5 ± 2.0	0.45 ± 0.04

^*^Means per layer orientation: 32.9 (0°), 19.3 (45°), and 22.7 (90°)

**Table 6:** Corrected densities and elastic moduli of the castor oil polymer for FEA models

Castor oil polymer application	Equivalent density (g/cm^3^)	Elastic modulus (MPa)
Coating the bamboo rods	1.032	825
Fill of the joints (trussed beam)	807	504
Fill of the joints (trussed cell and sandwich beam)		50.4
Adhesive layer	646	32.3

**Fig. 15:**
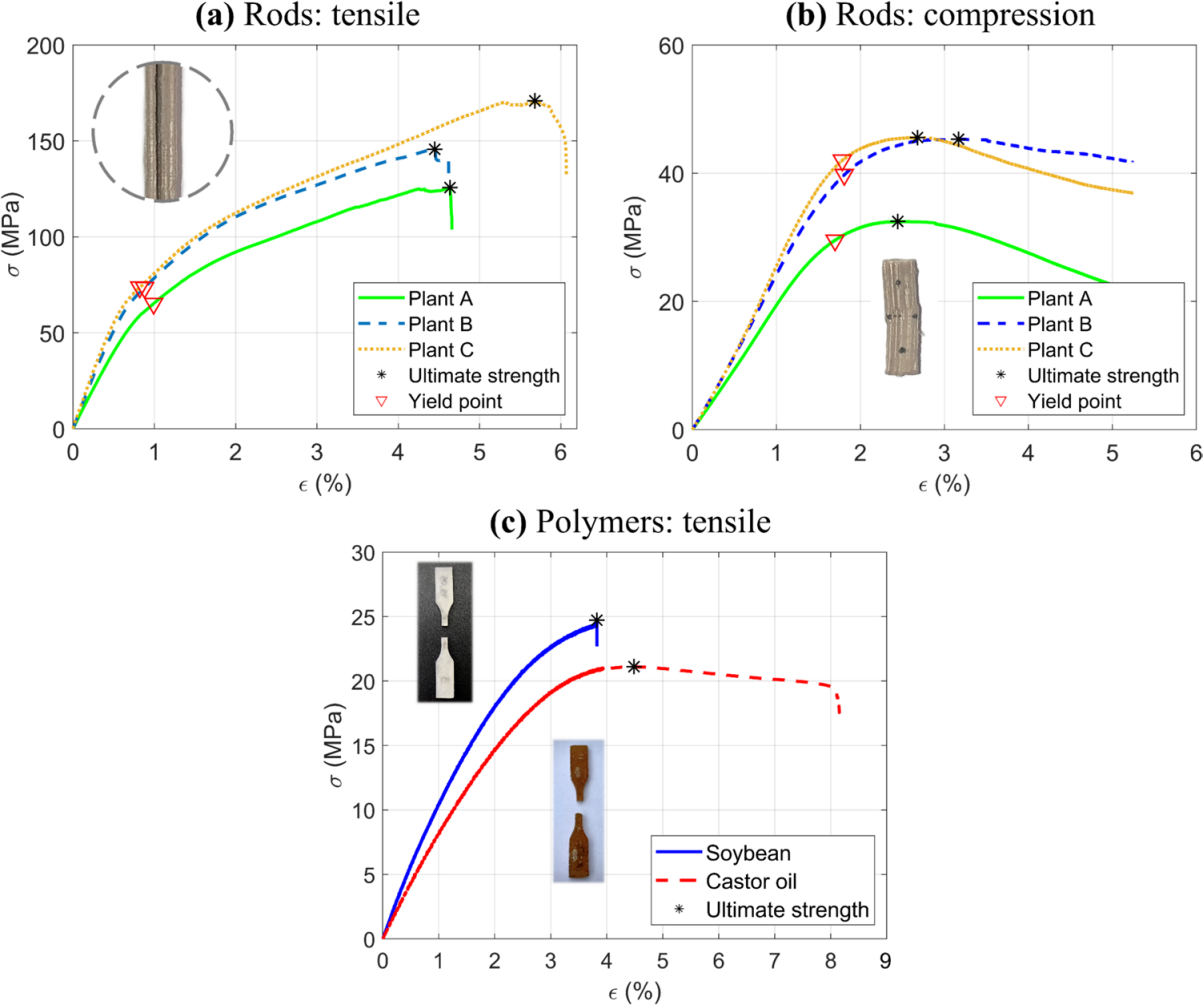
Stress–strain curves for mechanical assessment of the raw materials: **a** tensile of the bamboo rods, **b** compression of the bamboo rods, and **c** tensile of the soybean and castor oil polymers

The typical^3^ stress–strain behaviour of the raw materials is illustrated in Fig. 15. The tensile curves of the bamboo rods in Fig. 15a highlight the fracture as the sudden drop in the stress value between 4.5 and 6% strain, predominantly characterised by delamination of the bamboo structure [34]. In contrast, the compression behaviour, as shown in Fig. 15b, does not exhibit a distinct fracture point, instead resulting in the crushing of the specimen by about 3% strain [34]. Figure 15c compares the tensile behaviour of soybean and castor oil-based polymers, revealing similar elasticity and strength. However, the castor oil polymer demonstrates pronounced plastic deformation prior to failure, attributed to its elastomeric characteristics [64]. The tensile curves of both polymers depict a notable fracture point, at which the rupture occurs at the midsection of the specimen.

Evaluating the buckling behaviour of the rods by considering their compressive properties is crucial for understanding their structural stability. Equation (13), from Hibbler [82], allows for the estimation of the critical normal stress in a column just before it buckles (${\sigma }_{cr}$). The variable $E$ accounts for the modulus of elasticity of the component material, $L$ is the unsupported length of the column, and $K$ is the effective-length factor. Considering the conditions to which the rods are applied to the here-proposed trussed structures, $K$ is equal to 0.5 (fixed connections). The smallest radius of gyration of the column is $r=\sqrt{I/A}$, in which $I$ is the least moment of inertia and $A$ is the cross-sectional area. The smallest radius of gyration can be written as $r=\phi/4$ for the circular cross-section rods, being $\phi$ the diameter. As early depicted in Fig. 4a, in Sect. 2.1, the main members of the trussed structures have a diameter of 6 mm and 20 mm of unsupported length between the joints; whilst the auxiliary members are 4 mm in diameter and 27 mm in the unsupported length. By considering the critical case of Table 4, i.e., the lower elastic modulus for the bamboo rods under compression (2.11 GPa), the critical normal stresses are calculated in (14). The critical buckling stresses exceed the maximum compressive strength of the bamboo rods, indicating that buckling will not occur in the rods of the trussed structures prior to the members reaching the ultimate compressive strength.

13 $${\sigma }_{cr}=\frac{{\pi }^{2}E}{{\left(K\cdot{~}^{L}\!\left/ \!{~}_{r}\right.\right)}^{2}}$$

14 $${\sigma}_{cr}\approx \left.\begin{array}{l} {470} \, {\text{MPa}} \, {\text {for}} \, \phi =6 \, {\text{mm}} \, {\text {and}}\ L=20 {\text{mm}}\\ {115} \, {\text{MPa}} \, {\text{ for}} \, \phi =4 {\text{mm}} \, {\text {and}} L=27 {\text{mm}}\end{array} \right\vert E=2.11 {\text {GPa}}\ {\text {and}}\ K=0.5$$

#### Pullout, balsa wood, and lap-shear

**Fig. 16:**
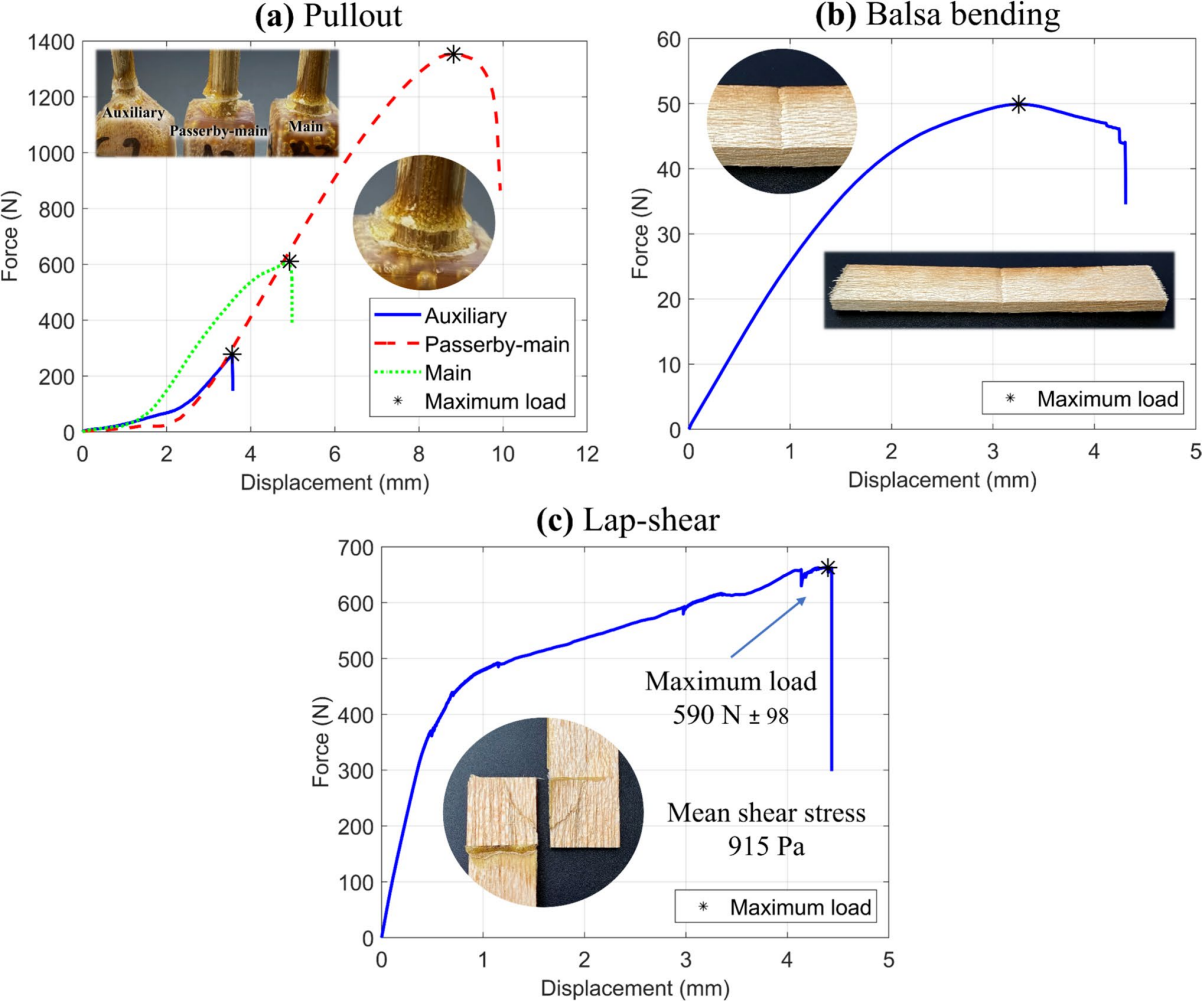
Force–displacement curves for mechanical assessment of the supplementary tests: **a** pullout for the rod-joints attachment, **b** three-point bending of the balsa wood, and **c** lap-shear for balsa and castor oil adhesivity

Figure 16a presents the force–displacement curves obtained from the pullout by a tensile test conducted on the attachments between the bamboo rods and the joints of the trussed structures, with detailed results provided in Table 7. The characteristic pullout failure, as the sudden drop in the force value, involves the extraction of the bamboo rod, accompanied by damage to both polymeric phases (soybean and castor oil). The maximum load capacities are 263 N for auxiliary members (corresponding to a rod axial stress of 20.9 MPa), 1,469 N for the passerby-main members (52.0 MPa of axial stress), and 610 N for the main members (axial stress of 21.6 MPa). All uniaxial stress values in the rods are below the yield stress under tensile loading, previously outlined in Table 4. Consequently, tensile loading in a truss member is more likely to result in pullout failure than non-linear deformation of the bamboo rod. Normalising the maximum load by the interface area of the rod that is embedded within the joins, as reported in Table 7, yields values ranging from approximately 3.5 MPa for auxiliary members to about 5.3 MPa for main members.

**Table 7:** Pullout performance for the attachment between the bamboo rods and polymeric joints

Pullout configuration	Maximum load (N)	Axial stress on the rod (MPa)	Stress based on the rod-fill interface area (MPa)
Auxiliary	263 ± 51	20.9 ± 4.1	3.49 ± 0.68
Passerby-main	1469 ± 182	52.0 ± 6.4	5.20 ± 0.64
Main	610 ± 45	21.6 ± 1.6	5.39 ± 0.40

Figure 16b illustrates the typical force–displacement curve for balsa wood under the three-point bending test, in which the failure, characterised by the drop of the load capacity, occurs by compression and crushing in the region in contact with the loading fixture. Table 8 summarises the experimentally measured physical and mechanical properties of the balsa wood: a bulk density of 0.1454 g/cm^3^, an elastic modulus along the grain direction of 2.50 GPa, and a flexural strength of 14.5 MPa. The curves proposed by Malek and Gibson [77] predict an elastic modulus of approximately 4.56 GPa for a density of 0.1454 g/cm^3^. Consequently, a correction factor is adopted, defined as the ratio between the here-measured experimental value (2.50 GPa) and the approximation by Malek and Gibson plots (4.56 GPa), yielding a factor of 0.548. Using this correction factor and the experimental density of 0.1454 g/cm^3^, the other elastic properties of interest for FEA modelling are estimated employing Malek and Gibson curves.

**Table 8:** Physical and mechanical properties of the balsa wood, as well as the engineering constants estimated by the Malek and Gibson curves [77], for application into the FEA models

Properties	Bulk density (g/cm^3^) 0.1454 ± 0.0044	Elastic modulus E1 (GPa) 2.50 ± 0.24	Flexural strength (MPa) 14.5 ± 1.6
→ Step 1: assessing the experimental data			
→ Step 2: estimating E1 through the Malek and Gibson curves to define the correction factor			
E1	4.56 GPa → Correction factor = 2.50/4.56 ≈ 0.548		
→ Step 3: estimating other data through the Malek and Gibson curves and by applying the correction factor			
E2	130 × 0.548 → ≈ 71.2 MPa		
E3	33.6 × 0.548 → ≈ 18.4 MPa		
Nu12	0		
Nu13	0.01		
Nu23	0.74		
G12	190 × 0.548 → ≈ 104 MPa		
G13	120 × 0.548 → ≈ 65.8 MPa		
G23	1.83 × 0.548 → ≈ 1.00 MPa		

Finally, Fig. 16c illustrates the characteristic force–displacement behaviour observed during lap-shear tests, conducted to rigorously evaluate the interfacial adhesion between the castor oil-based polymer and the balsa wood lamina. The results consistently demonstrate shear rupture occurring within the balsa wood across all specimens, at a maximum load of approximately 590 N. This corresponds to a mean shear stress of 915 Pa. These findings suggest that, in the context of the proposed sandwich beam under bending, the failure is prone to occur in the balsa structure rather than by delamination at the adhesive interface. This emphasises the adequacy of the castor oil polymer bonding performance for such structural applications.

### Metastructure trussed cell

#### Physical and mechanical characteristics

Table 9 shows the physical properties of the metastructure trussed cell, detailing the means and standard deviation of the three distinct replicates, made with rods from bamboo plants A, B, and C. The overall means and their standard deviations are based solely on the mean values of each replicate, and cell B represents the typical mean response. The trussed cells have a mass of about 30 g, and their global size remains relatively consistent with the initial design of 50 × 50 × 50 mm^3^, with slight variations. This dimensional variation may be attributed to the expansion of the castor oil polymer during the curing process, which marginally influences the dimensional tolerances of the trussed cell. The effective material area at the midsection of the trussed cell and the bulk volume of material in the whole structure, as estimated through the CAD model, are nearly 215 mm^2^ and 40 cm^3^; which represent about 9% of the equivalent area (≈ 50 × 50 mm^2^) and 35% of the equivalent volume (≈ 125 cm^3^). These characteristics concerning mass and dimensions of the trussed cells provide bulk and equivalent densities of about 0.80 g/cm^3^ and 0.23 g/cm^3^, respectively. Furthermore, the mass composition analysis reveals that bamboo rods compound 31% of the overall weight of the metastructure, whilst soybean and castor oil polymers contribute 16% and 53%, respectively, Therefore, the trussed cell is 69% composed of bio-based polymer material and 31% by natural composite rods extracted from the giant bamboo.

**Table 9:** Physical properties of the metastructure trussed cell

Trussed cell	Mass (g)	Mean global size*: height × (homogenised cross-sectional area) (mm^3^)	Effective cross-sectional area from CAD model (mm^2^)	Bulk volume from CAD model (cm^3^)	Equivalent volume (cm^3^)	Bulk density (g/cm^3^)	Equivalent density (g/cm^3^)
Cell A	27.91 ± 0.74	51.05 × (49.90 × 49.77)	214.7	36.90	126.8 ± 1.6	0.7564 ± 0.0200	0.2201 ± 0.0054
Cell B	30.18 ± 0.43	50.84 × (50.09 × 50.12)			127.6 ± 1.6	0.8179 ± 0.0117	0.2365 ± 0.0033
Cell C	30.38 ± 0.73	49.77 × (50.23 × 50.72)			126.8 ± 0.4	0.8233 ± 0.0198	0.2396 ± 0.0059
Overall mean	29.49 ± 1.37	50.55 × (50.07 × 50.20)			127.1 ± 0.4	0.7992 ± 0.0372	0.2321 ± 0.0105

^*^Maximum deviation of ± 0.34 mm per dimension

**Fig. 17:**
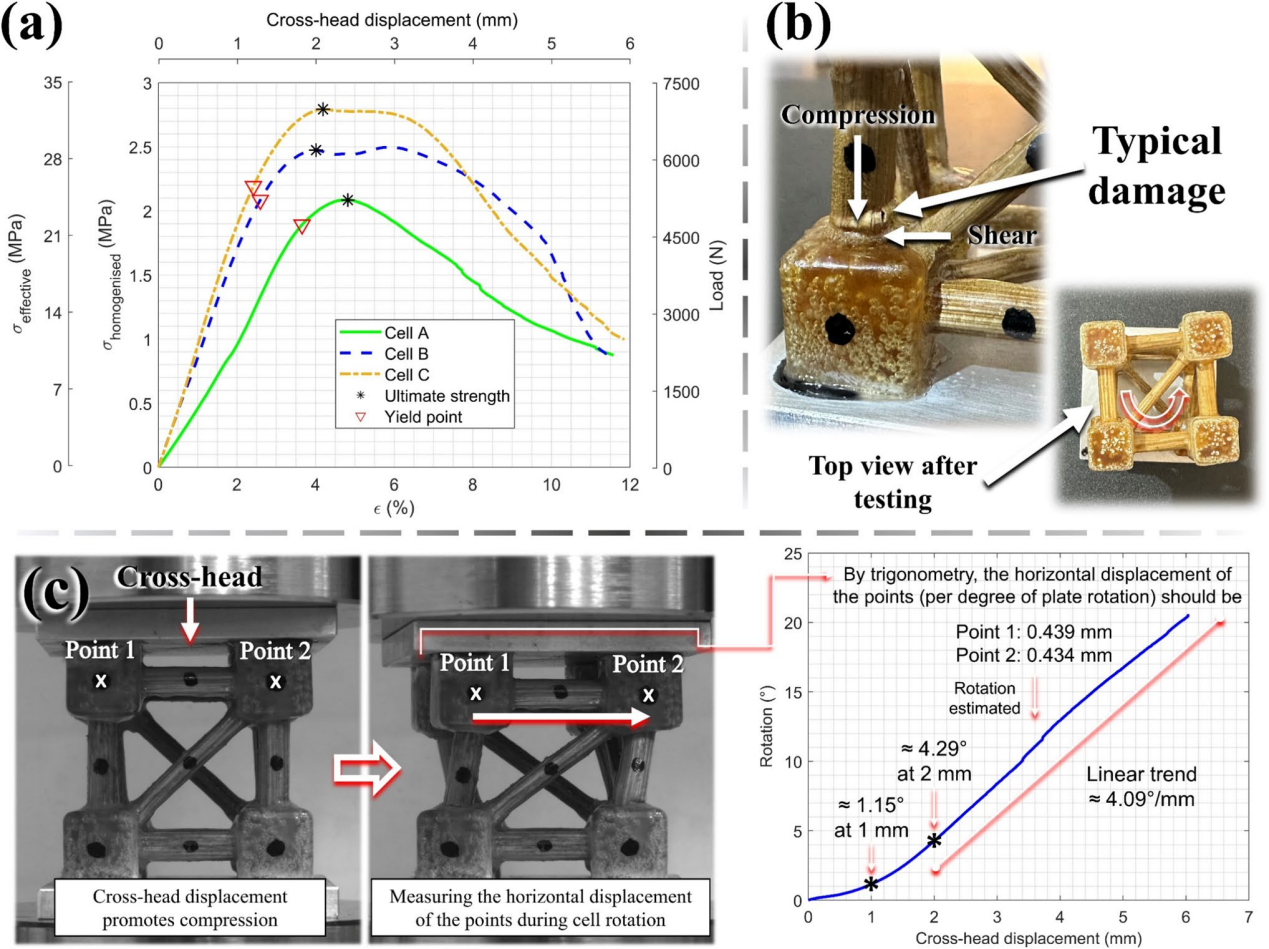
Mechanical characteristics of the trussed cell: **a** force–displacement curves under compression, **b** typical damage observed in the truss members, and **c** insights into the rotational behaviour of the metastructure

Table 10 presents the compressive properties of the metastructure trussed cell derived from force–displacement data and stress values calculated using the effective cross-sectional area. Notably, the maturity of the plants from which the rods are extracted (A, B, or C) has a significant influence on the mechanical properties of the trussed cells. The overall mean values of the compressive properties emphasise the consistency of cell B’s performance as a representative sample, making the rods extracted from plant B a reliable benchmark for the fabrication of the trussed structures. The mean maximum load sustained by the trussed cell under compressive loading is approximately 6,200 N, which increases up to nearly 7,100 N when incorporating bamboo rods harvested from plants at advanced stages of maturity (cell C). As illustrated in Fig. 17a, the maximum load capacity of the trussed cell is achieved at an overall displacement between 2 and 2.5 mm. Beyond this point, the response exhibits behaviour akin to that observed in bamboo rods subjected to compression, as previously shown in Fig. 15b. Notably, the force–displacement curve does not feature a distinct fracture point, which should be characterised by an abrupt drop in the force value. Instead, a gradual decline in the measured force is observed as the trussed cell undergoes rotation, with the four main member rods aligned to compressive load experiencing progressive crushing until damage occurs due to compression and shear efforts—refer to Fig. 17b.

**Table 10:** Compressive properties of the metastructure trussed cell. Stress data based on the effective cross-sectional area

Trussed cell	Maximum load (N)	Maximum displacement (mm)	Slope* (N/mm)	Rotation** (°/mm)	Elastic modulus (GPa)	Yield stress (MPa)	Yield strain (%)	Ultimate stress (MPa)	Ultimate strain (%)	Modulus of resilience (μJ/mm^3^)	Modulus of toughness (μJ/mm^3^)
Cell A	5176 ± 243	2.462 ± 0.032	3176 ± 235	1.79 ± 0.14	0.7553 ± 0.0559	21.89 ± 0.50	3.657 ± 0.192	24.11 ± 1.13	4.823 ± 0.062	387.5 ± 9.3	660.2 ± 35.0
Cell B	6210 ± 266	2.040 ± 0.071	4350 ± 196	1.15 ± 0.10	1.030 ± 0.046	24.37 ± 0.46	2.594 ± 0.194	28.92 ± 1.24	4.012 ± 0.140	333.6 ± 8.4	722.1 ± 30.3
Cell C	7115 ± 212	2.086 ± 0.053	5212 ± 276	1.01 ± 0.11	1.208 ± 0.064	26.01 ± 1.00	2.418 ± 0.144	33.14 ± 0.99	4.191 ± 0.107	326.6 ± 6.6	870.5 ± 14.7
Overall mean	6167 ± 970	2.196 ± 0.232	4246 ± 1022	1.32 ± 0.42	0.9978 ± 0.2281	24.09 ± 2.07	2.890 ± 0.670	28.72 ± 4.52	4.342 ± 0.426	349.2 ± 33.3	750.9 ± 108.1

^*^At the linear portion of the curve, up to a displacement of 1 mm. **Instantaneous rate at 1 mm of displacement

Referring back to Fig. 17 (a), it is evident that the linear behaviour in the force–displacement curves occurs up to nearly 1 mm, with a mean slope of approximately 4200 N/mm (Table 10). Figure 17c highlights the rotational behaviour of the metastructure under compression, with the typical curve (cell B) indicated. Within the linear regime of the structure, the instantaneous rotation for 1 mm of cross-head displacement is 1.15°. During the yielding phase up to the point of maximum loading (approximately 2 mm), the rotational behaviour transitions through a non-linear regime, reaching a rotation of approximately 4°. Beyond this, the rotational behaviour stabilises at nearly 4° per millimetre of cross-head displacement, exhibiting a linear trend. The structure undergoes a total rotation of about 20° for a cross-head displacement of 6 mm. Figure 17b depicts the permanent rotation in the top view of the specimen following the testing procedure; and, notably, a substantial portion of the rotation is recovered upon removal of the sample from the testing machine. Considering all the plant maturities employed for cell fabrication (A, B, and C), the overall mean instantaneous rotation for 1 mm of cross-head displacement is about 1.32° (see Table 10). The metastructure trussed cells exhibit, approximately, the following additional effective properties: an elastic modulus of 1 GPa; yield stress and strain of 24 MPa and 3%; ultimate stress and strain of 29 MPa and 4%; modulus of resilience of 349 μJ/mm^3^; and modulus of toughness (up to the maximum load) of 751 μJ/mm^3^. These properties indicate a well-balanced combination of stiffness, strength, and energy absorption capacity. Finally, it is important to highlight that within the elastic regime, the transverse dimensions of the trussed cell remain unchanged. This indicates that, for small displacements under compressive loading, the cell deforms primarily through uniform rotation rather than lateral expansion or contraction. As a result, the structure exhibits an equivalent Poisson ratio of zero, meaning that axial compression does not induce any transverse global deformation.

Table 11 shows the compressive properties of the metastructure trussed cell, with stress data based on the homogenised cross-sectional area. The structure exhibits, roughly, the following homogenised properties: elastic modulus of 85 MPa, yield stress of 2.1 MPa, ultimate stress of 2.5 MPa, modulus of resilience of 30 μJ/mm^3^, and modulus of toughness of 64 μJ/mm^3^. Finally, Table 12 depicts the mean specific properties of the trussed cells, encompassing load capacity, rigidity, and strength. The specific load capacity is determined by dividing the maximum load supported under compression by the mass of the structure. Specific rigidity and strength are calculated by dividing the elastic modulus and ultimate stress, respectively, by the structure’s density (both bulk and equivalent). The overall mean specific load capacity is approximately 208.5 N/g, which indicates that the metastructure trussed cell can support 21,258 times its mass under compressive loading. Considering the effective stress data and bulk density, the specific elastic modulus of the cell is about 1.2 GPa∙cm^3^/g, and its specific ultimate stress is nearly 36 MPa∙cm^3^/g. Using the homogenised stress data and equivalent density, the specific elastic modulus and strength are, respectively, approximately 365 MPa∙cm^3^/g and almost 11 MPa∙cm^3^/g.

**Table 11:** Compressive properties of the metastructure trussed cell. Stress data based on the homogenised cross-sectional area

Trussed cell	Elastic modulus (MPa)	Yield stress (MPa)	Ultimate stress (MPa)	Modulus of resilience (μJ/mm^3^)	Modulus of toughness (μJ/mm^3^)
Cell A	65.30 ± 4.83	1.892 ± 0.043	2.084 ± 0.098	33.49 ± 0.80	57.07 ± 3.03
Cell B	88.11 ± 3.94	2.084 ± 0.039	2.474 ± 0.106	28.53 ± 0.72	61.76 ± 2.59
Cell C	101.8 ± 5.4	2.192 ± 0.084	2.793 ± 0.083	27.52 ± 0.52	73.36 ± 1.24
Overall mean	85.08 ± 18.45	2.056 ± 0.152	2.450 ± 0.355	29.85 ± 3.20	64.06 ± 8.38

**Table 12:** Mean specific compressive properties of the metastructure trussed cell: load capacity, rigidity, and strength

	By mass		By effective stress and bulk density		By homogenised stress and equivalent density		
Trussed cell	Specific maximum load (N/g)	Specific maximum load (g/g)	Specific elastic modulus (GPa∙cm^3^/g)	Specific ultimate stress (MPa∙cm^3^/g)	Specific elastic modulus (MPa∙cm^3^/g)	Specific ultimate stress (MPa∙cm^3^/g)	
Cell A	185.5	18,911	0.9985	31.87	296.7	9.468	
Cell B	205.8	20,982	1.259	35.36	372.6	10.46	
Cell C	234.2	23,882	1.467	40.25	424.9	11.66	
Overall mean	208.5 ± 24.5	21,258 ± 2497	1.242 ± 0.235	35.83 ± 4.21	364.7 ± 64.5	10.53 ± 1.10	

#### FEA insights

**Fig. 18:**
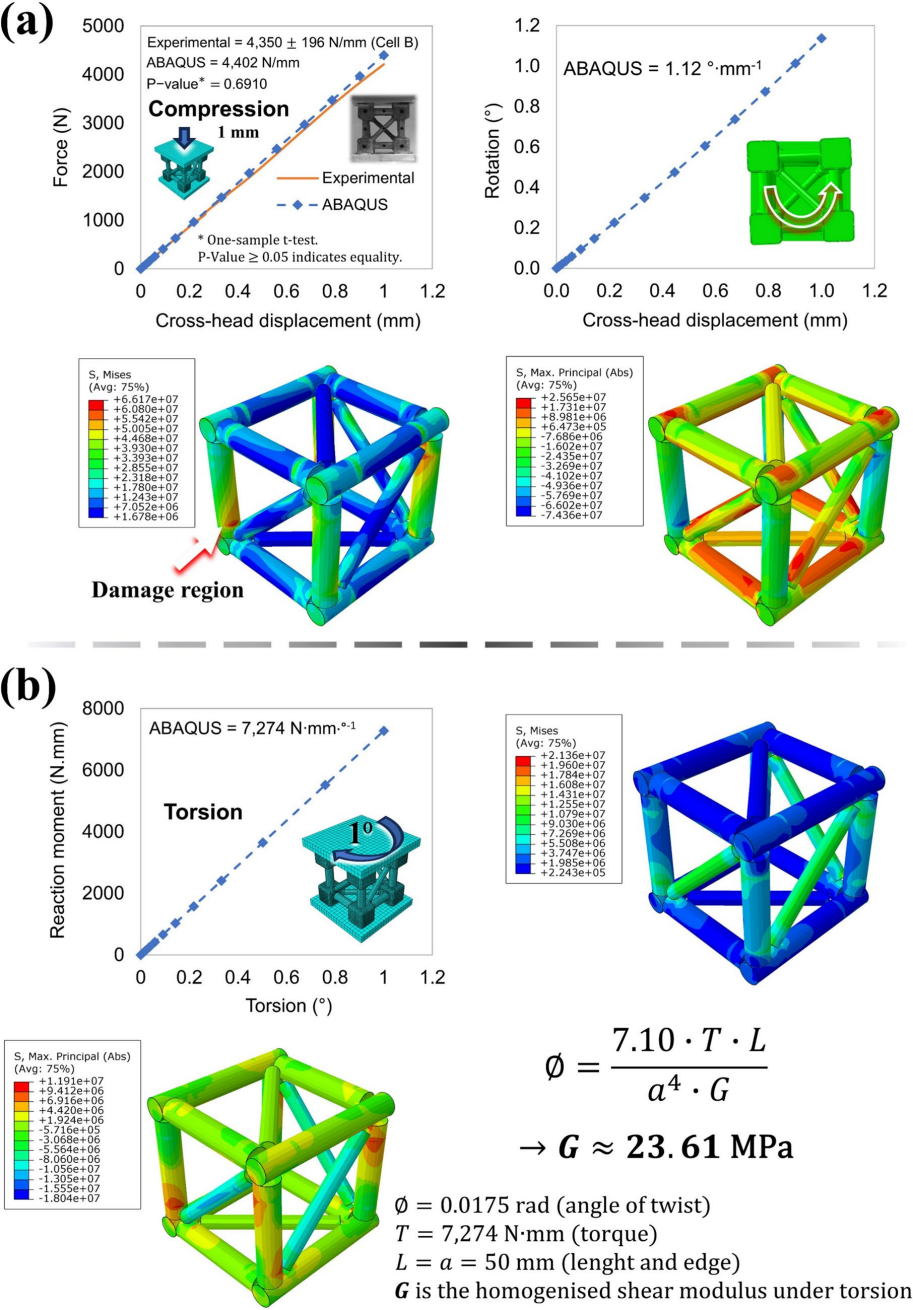
FEA insights on the metastructure trussed cell: **a** validation of the model under compression and evaluation of stress distribution in the rods; and **b** results on the torsion assessment

Figure 18a illustrates the comparison of the force–displacement curves within the linear regime (up to 1 mm) between the experimental and simulated approaches, validating the FEA model. The numerical model takes into account the properties of the rods extracted from plant B (the representative one). The one-sample t-test is employed to assess whether the mean value of the experimental sample significantly deviates from a specified value. In this case, the test is applied to compare the experimental data with the simulated result. The P-value greater than 0.05 suggests that there is no statistically significant difference between the experimental and simulated values. The rotation of the metastructure, measured through the FEA model, is 1.12° for 1 mm of cross-head displacement, which is consistent with the experimental value of 1.15° previously shown in Fig. 17c. The stress distribution in the rods is illustrated from two perspectives: (i) the von Mises and (ii) the absolute maximal principal. It is possible to observe the critical stresses concentrated in the region where damage occurs, as previously reported in Fig. 17b. The absence of global transverse deformations in the elastic regime (equivalent zero Poisson ratio), as depicted in the experimental approach, is also noticed in the FEA model for compression of the trussed cell.

Based on the validated FEA model, Fig. 18b presents the reaction moment of the metastructure trussed cell for torsion with an angle of twist of 1°, which results in a torque of 7,274 N∙mm. The stress distribution in the rods when the cell is subjected to torsion is also shown, with the most critical values occurring in the auxiliary members that bear the compression load when torsion is applied to the trussed cell. Using the analytical equations addressed in [82], a homogenised shear modulus under torsion of approximately 24 MPa can be estimated.

### Trussed and sandwich beams

#### Physical and mechanical attributes

Table 13 summarises the physical properties of the trussed and sandwich beams, presenting the means and standard deviations from the three specimens of each structure fabricated using rods sourced from plant B. The trussed beam exhibits a mass of approximately 189 g, with overall dimensions remaining closely aligned to the initial design specifications of 400 × 50 × 50 mm^3^, subject to minor deviations. Similarly, the sandwich beam has a mass of roughly 301 g, with dimensions consistent with its original design of 406 × 54 × 60 mm^3^.

**Table 13:** Physical properties of the trussed and sandwich beams

Structure	Mass (g)	Mean global size*: length × width × thickness (mm^3^)	Bulk volume from CAD model (cm^3^)	Equivalent volume (cm^3^)	Bulk density (g/cm^3^)	Equivalent density (g/cm^3^)
Trussed beam	189.0 ± 3.5	399.00 × 50.22 × 50.31	244.0	1008 ± 16	0.7746 ± 0.0144	0.1875 ± 0.0064
Sandwich beam	300.5 ± 6.4	406.02 × 54.01 × 64.03	593.0	1404 ± 18	0.5067 ± 0.0108	0.2141 ± 0.0052
Mass composition (wt%)						
Trussed beam			Bamboo rods (40% ± 2%), soybean polymer (15% ± 2%), castor oil polymer (45% ± 4%)			
Sandwich beam		Balsa wood (11% ± 1%), Bamboo rods (25% ± 3%), soybean polymer (10% ± 1%), castor oil polymer (55% ± 5%)				

^*^Maximum deviation of ± 0.41 mm per dimension

The expansion of the castor oil polyurethane during the curing process appears to provide a minimal effect on the dimensional stability of the trussed beam, however, it does affect the thickness of the sandwich beam by expanding the adhesive layer that bonds the core and the skins (as previously discussed in Sect. 2.2.3). The volumetric analysis, based on the CAD models, estimates the total bulk material volume to be approximately 244 cm^3^ for the trussed beam and 593 cm^3^ for the sandwich beam. These volumetric and mass characteristics yield to the trussed beam a bulk and equivalent densities of about 0.77 g/cm^3^ and 0.19 g/cm^3^, respectively. Notably, these densities are slightly lower than those reported for the trussed cell – refer to Table 9. This minor discrepancy arises because the trussed beam is assembled through a series association of trussed cells so that the adjacent cells share common joints. Consequently, the polymer mass fraction within the trussed beam is reduced compared to that in individual trussed cells, leading to lower densities, as the polymeric phases are inherently denser than the bamboo rods, as previously depicted in Tables 4 and 5. For the sandwich beam, the bulk and equivalent densities are approximately 0.51 g/cm^3^ and 0.21 g/cm^3^, respectively. An analysis of the mass composition reveals that the trussed beam comprises 40% natural composite rods extracted from giant bamboo and 60% polymeric material, further subdivided into 15% soybean-based polymer and 45% castor oil-based polymer. In contrast, the mass composition of the sandwich beam consists of 11% balsa wood, 25% bamboo rods, and 65% polymeric material, of which 10% is soybean-based and 55% is castor oil-based.

**Fig. 19:**
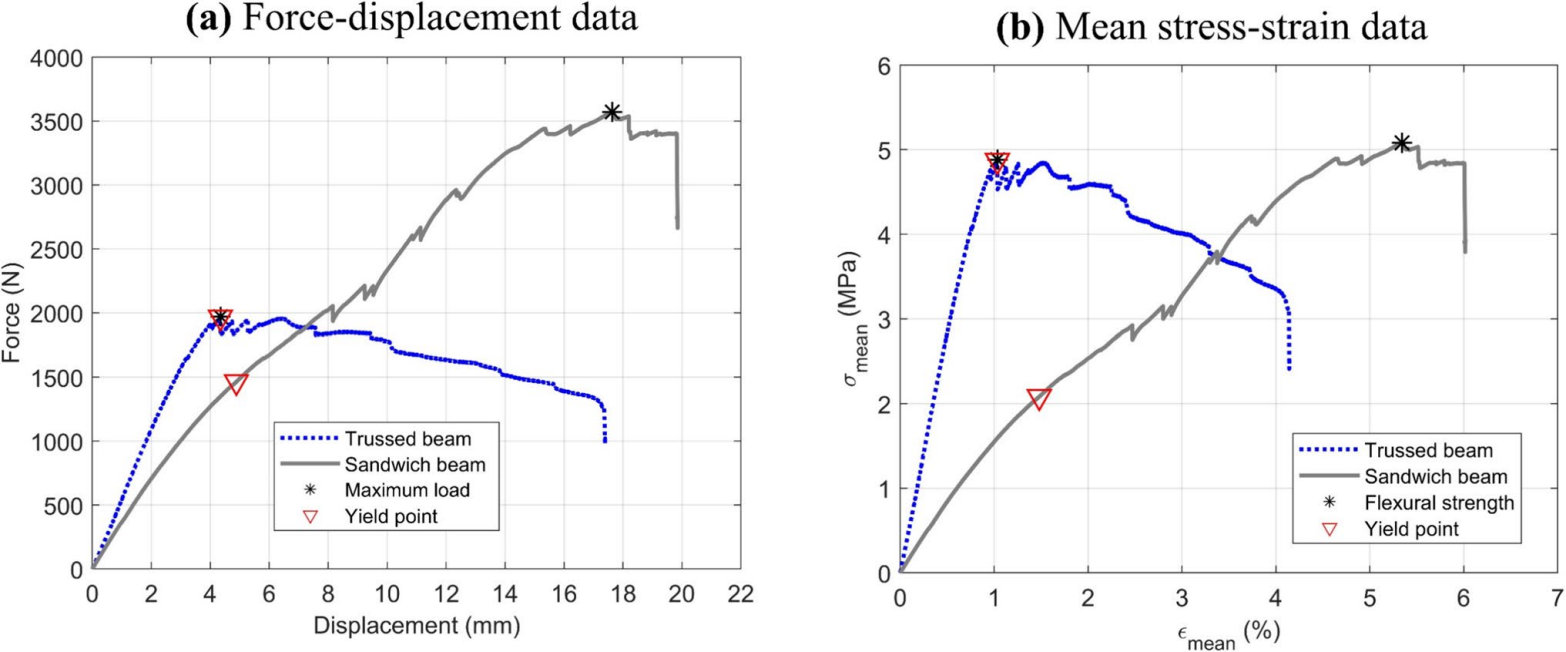
Four-point bending curves for the trussed and sandwich beams: **a** force–displacement data, and **b** mean stress–strain data

Table 14 highlights the mechanical properties of the beams under four-point bending tests, encompassing force–displacement and mean stress–strain data, with the corresponding typical curves illustrated in Fig. 19. It is noteworthy that the central deflection of the beams, measured using the Video Gauge™, is found to be identical to the cross-head displacement of the test. The force–displacement behaviour depicted in Fig. 19a reveals that both beams exhibit a linear response up to approximately 2 mm. Beyond this point, a slight deviation from linearity is observed (though the response remains nearly linear) up to approximately 4 mm, where the yield point emerges.

**Table 14:** Four-point bending properties of the trussed and sandwich beams

Structure	Slope* (N/mm)	Maximum load (N)	Maximum displacement (mm)	Maximum bending moment (kN∙mm)	Experimental flexural modulus (MPa)	Yield mean stress (MPa)	Yield mean strain (%)	Ultimate mean stress (MPa)	Ultimate mean strain (%)	Modulus of resilience (μJ/mm^3^)	Modulus of toughness (μJ/mm^3^)
Trussed beam	537.7 ± 33.1	1969 ± 234	4.348 ± 0.569	103.4 ± 12.3	587.3 ± 36.2	4.870 ± 0.300	1.034 ± 0.095	4.880 ± 0.591	1.035 ± 0.136	28.52 ± 1.93	157.8 ± 20.6
Sandwich beam	369.6 ± 26.1	3571 ± 583	17.63 ± 2.09	187.5 ± 30.6	159.9 ± 11.3	2.083 ± 0.340	1.480 ± 0.136	5.081 ± 0.803	5.343 ± 0.634	16.98 ± 1.26	193.4 ± 27.1

^*^At the linear portion of the curve, up to a displacement of 2 mm

In the linear portion of the curves, as depicted in Table 14, the trussed beam exhibits a force–displacement slope under bending of approximately 538 N/mm, while the sandwich beam displays a value near 370 N/mm. Although it is typically expected that a sandwich structure would present a steeper slope in the linear portion of the curve, due to the anticipated higher flexural stiffness associated with sandwich-type configurations, the lower value observed for the sandwich beam can be attributed to the occurrence of shear deformations and due to the balsa wood intrinsic anisotropy, as further detailed in the text (Sect. 3.3.2). The trussed beam withstands a maximum load of nearly 2,000 N for a central deflection of approximately 4 mm. It is noteworthy that the yield point and the maximum load of the trussed beam are virtually coincident—refer to Fig. 19a. This behaviour is attributed to the type of failure mechanism that occurs in the structure, which is also properly elucidated soon through a failure analysis. The sandwich beam also exhibits yielding at a deflection close to 4 mm; however, it continues to deform, supporting a load of almost 3600 N for a displacement of around 17.5 mm. These maximum load values result in a maximum bending moment of approximately 103 kN∙mm for the trussed beam and 188 kN∙mm for the sandwich beam.

The homogenised mechanical properties of the beams, as depicted in Table 14 through the mean stress–strain data illustrated in Fig. 19b, provide additional insights into the mechanical response of the beam structures under four-point bending. The experimental flexural modulus aligns with the slope of the force–displacement curve, with the trussed beam exhibiting a higher measurement compared to the sandwich beam (587.3 MPa in contrast to 159.9 MPa). Yield and ultimate stresses are about 5 MPa for the trussed beam, at a mean strain of nearly 1%. The ultimate stress of the sandwich beam is similar to the trussed beam (~ 5 MPa), but at a mean strain rate of about 5%. The yield mean stress of the sandwich beam is about 2 MPa for a yield mean strain of almost 1.5%. At the yield point, reaching a mean stress value equivalent to the ultimate mean stress of the sandwich beam, the trussed beam demonstrates a higher modulus of resilience than the sandwich configuration (~ 29 μJ/mm^3^ compared to ~ 17 μJ/mm^3^). The ultimate fracture of the structures is characterised by the explicit sudden drop in the load value, as seen in the curves presented in Fig. 19. For the trussed beam, it occurs at a displacement of approximately 17 mm (~ 4% of mean strain), while the ultimate fracture of the sandwich beam takes place at a test displacement of around 20 mm (~ 6% of mean strain). Consequently, the energy absorbed up to fracture is greater for the sandwich beam, with a modulus of toughness of approximately 193 μJ/mm^3^ compared to ~ 158 μJ/mm^3^ for the trussed beam.

**Fig. 20:**
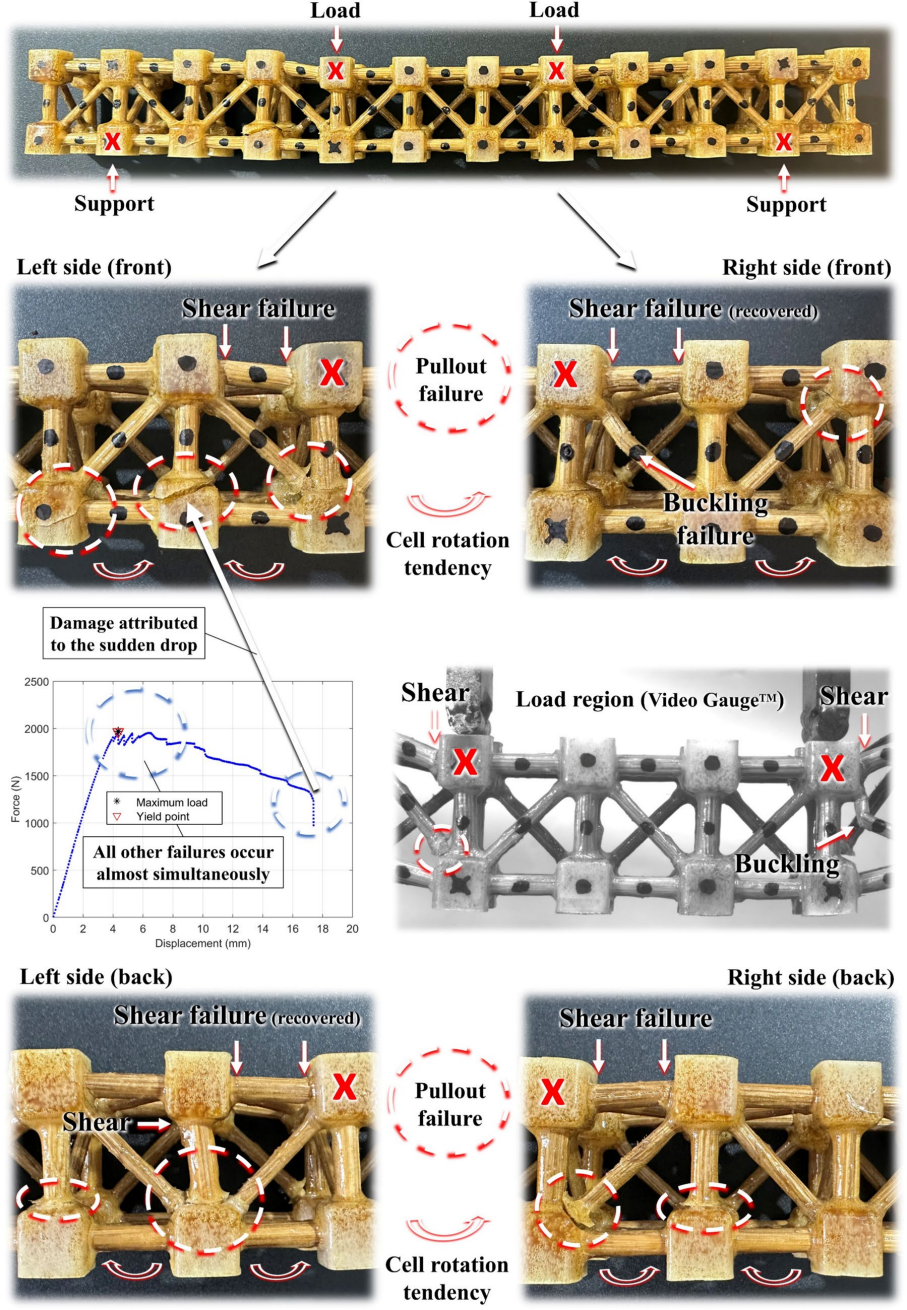
Failure analysis of the trussed beam

Figure 20 provides insights into the failure analysis of the trussed beam. The deformed structure post-testing is highlighted, with an X-mark indicating the joints in contact with the support and loading apparatus. An enlarged view accentuates the left and right portions of the frontal perspective, where the observed damages are primarily localised. Owing to the structural symmetry of the trussed beam, the geometric configuration of the left-side-front view aligns with that of the right-side-back view, while the right-side-front view mirrors the left-side-back view. The damage predominantly manifests in the third and fourth cells from the extremities, consisting chiefly of pullout failures, underscoring that the critical members are under tensile loading. As previously described in Sect. 3.1.3, the pullout failures typically precede any non-linear deformation of the bamboo rods. This observation explains why, in the trussed beam configuration, the yield point coincides almost precisely with the maximum load. Pullouts generally occur while the structure remains within the linear elastic regime, resulting in the maximum load aligning with the onset of yielding. Concerning the ultimate failure of the trussed beam, the characteristic damage responsible for the sudden drop in the loading curve is evident in the left-side-front view: a critical pullout culminating in a full rupture of the joint structure. Near the loading region, shear failure of the longer main bamboo rod can be observed, along with occasional buckling of an auxiliary member subjected to compressive forces at the joint experiencing loading by the testing tool. Additionally, just as occurred for the trussed cell under compression, partial recovery of deformation post-testing is noticeable once the trussed beam specimen is removed from the testing machine. This behaviour is attributed to the fibrous nature of bamboo rods and the elastomeric characteristics of the bio-based castor oil polymer.

**Fig. 21:**
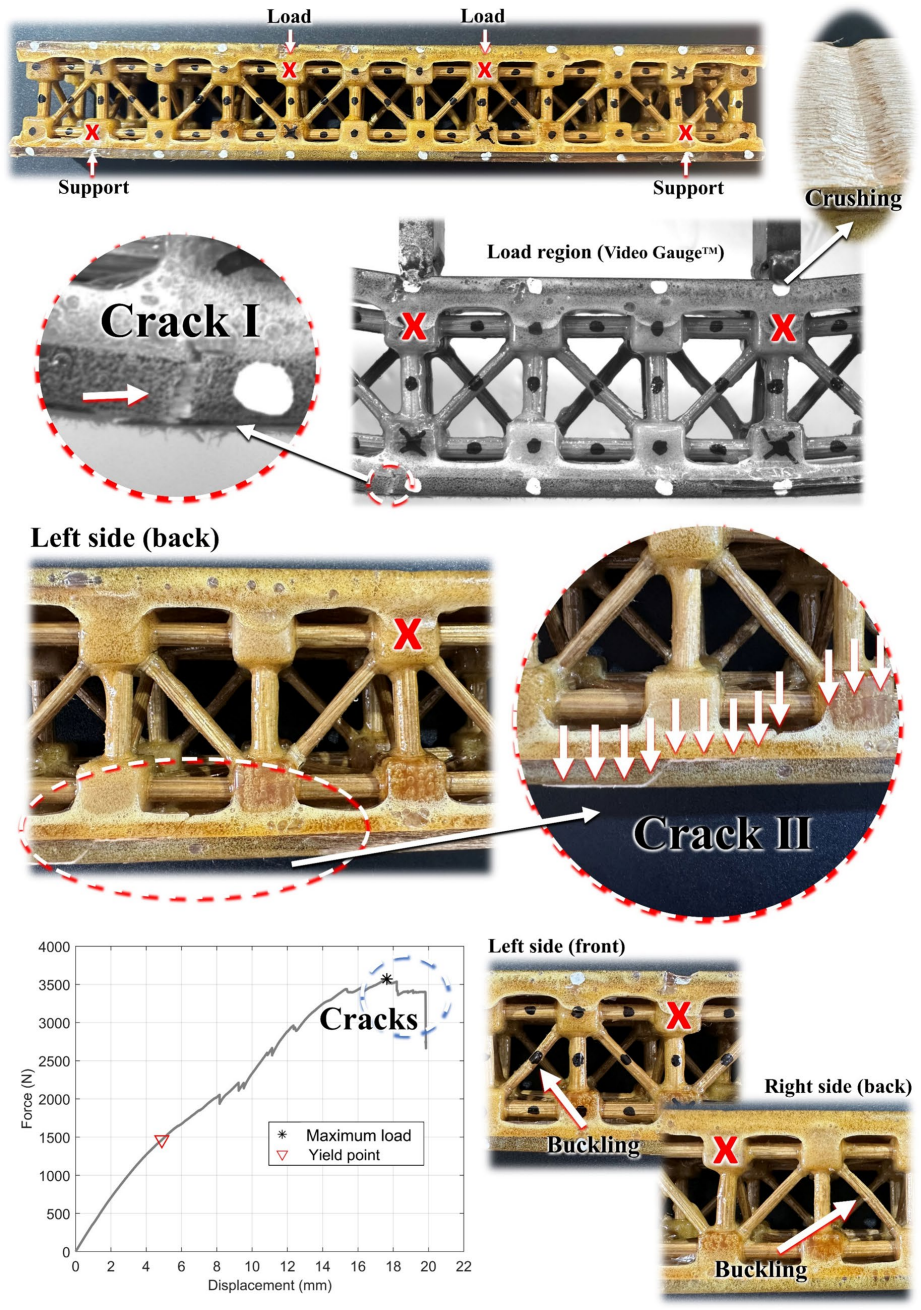
Failure analysis of the sandwich beam

Figure 21 illustrates the failure mechanisms observed in the sandwich beam. Localised crushing damage occurs in the skins at the regions in contact with the support and loading apparatus, attributable to the low transverse stiffness of balsa wood – refer to Table 8. However, the damage leading to the ultimate failure comprises combined cracking phenomena. These include a crack initiating in the bottom skin near the loading region (crack I) and an additional crack propagating within the third and fourth cells from the beam’s extremities (crack II). Crack II propagates from the balsa wood skin through the castor oil adhesive layer and into the bi-component joint. These cracks primarily occur due to tensile stresses in the bottom skin, which is characteristic of sandwich structures under bending loads. The rotational effect of the metastructure cells may contribute to this phenomenon, potentially inducing a certain degree of torsion.

Still assessing Fig. 21, in contrast to the trussed beam, the absence of pullouts is evident on the sandwich beam. The sandwich configuration effectively distributes the efforts across all the joints, which experience greater deformation (as previously discussed through the need to perform the correction of the elastic modulus of the castor oil joint fill material for the FEA models in Sect. 2.4). However, the relative displacement of the joints concerning the rods is significantly reduced in the sandwich beam, which contributes to limiting the pullout effect, enabling the structure to withstand higher loads. There is a serrated behaviour observed in the loading curves between the yield point and the maximum load. This phenomenon has been noted and reported in the mechanical analysis of natural composite rods extracted from giant bamboo when subjected to tensile testing [34], and it has been attributed to the stick–slip behaviour of the bamboo fibres within the lignocellulosic matrix. Concerning the sandwich structure, these serrations may be ascribed to a similar stick–slip effect occurring between the rods and the adhesive at the joints. While this interaction does not appear to be strong enough to induce complete pullout failure, it still may manifest as localised micro-slips within the bonded regions. Regarding compressive damage, a minimal occurrence of buckling is observed in the auxiliary members within the third cell from the beam extremities (see left-side-front and right-side-back views).

#### Specific properties and the RJS method results

Table 15 presents the mean specific properties of the trussed and sandwich beams under four-point bending, including the maximum moment, rigidity, and strength. The specific maximum bending moment is obtained by dividing the maximum bending moment by the mass of the structure within the support span length. To calculate this mass (${m}_{S}$), it is assumed that the mass distribution within the beams is approximately uniform along the longitudinal axis, with the relation ${m}_{S}=(S/{l}_{b})\cdot {m}_{b}$ being employed, where $S$ represents the support span length, ${l}_{b}$ denotes the length of the beam, and ${m}_{b}$ is the total mass of the beam. Specific rigidity and strength are determined by dividing the experimental flexural modulus and ultimate mean stress, respectively, by the density of the structures (both bulk and equivalent). The specific maximum bending moment of the trussed beam is about 695 N∙mm/g, compared to ~ 804 N∙mm/g for the sandwich beam. The sandwich beam also outperforms the trussed beam in terms of specific flexural strength normalised by the bulk density: ~ 10 MPa∙cm^3^/g compared to ~ 6 MPa∙cm^3^/g. Nevertheless, the opposite occurs when the equivalent density is taken into account: ~ 26 MPa∙cm^3^/g for the trussed beam compared to ~ 24 MPa∙cm^3^/g of the sandwich beam. In contrast, the specific experimental flexural moduli of the trussed beam are higher than the ones for the sandwich beam: ~ 758 MPa∙cm^3^/g compared to ~ 316 MPa∙cm^3^/g, based on the bulk density; and 3,132 MPa∙cm^3^/g compared to ~ 747 MPa∙cm^3^/g, based on the equivalent density.

**Table 15:** Mean specific four-point bending properties of trussed and sandwich beams: maximum moment, rigidity, and strength

	By mass	By bulk density		By equivalent density		
Structure	Specific maximum bending moment* (N∙mm/g)	Specific experimental flexural modulus (MPa∙cm^3^/g)	Specific ultimate mean stress (MPa∙cm^3^/g)	Specific experimental flexural modulus (MPa∙cm^3^/g)	Specific ultimate mean stress (MPa∙cm^3^/g)	
Trussed beam	694.9	758.2	6.300	3132	26.03	
Sandwich beam	804.4	315.5	10.03	746.7	23.73	

^*^Normalised by the mass of material within the support span length in bending

Based on RJS equations [56], Table 16 shows that the flexural modulus of the sandwich beam under pure bending should be 1.370 GPa, which is approximately 8.6 times higher than its experimentally measured flexural modulus (~ 160 MPa), and 2.3 times greater than that of the core material (trussed beam, ~ 587 MPa). The flexural stiffness of the sandwich beam is 1.618 × 10^9^ N∙mm^2^. The reduced experimental flexural modulus of the sandwich beam, representing only ~ 12% of pure bending, can be attributed to two phenomena: (i) the occurrence of shear deformations within the core, as reported by the RJS Method; and (ii) the high anisotropy of the balsa wood skins, which has also been identified by the RJS Method as a potential factor contributing to a lower experimental flexural modulus relative to the theoretical prediction. The crushing of the balsa wood, due to its low transverse stiffness (~ 18 MPa, as previously shown in Table 8), corroborates the reduction of the slope of the force–displacement curve and the subsequent decrease in the experimental flexural modulus of the sandwich beam. This effect introduces an error in the estimation performed through the RJS equations for the shear stiffness of the sandwich beam (2.207 × 10^4^ N) and for the core shear modulus (~ 6 MPa). To achieve 95% pure bending in this sandwich beam, a span length of approximately 3600 mm would be required. Such a span length would be impractical since, as previously discussed in Sect. 2.2.1, beams longer than 400 mm would require bamboo rods containing the nodes of the plant, which would likely reduce the rigidity and strength of the trussed beam core. Multiple trussed beams of 400 mm in length could be assembled in series to achieve such a long span; however, the stiffness and strength of the resulting assembly would not be equivalent to that of a single continuous beam. Additionally, assembling a beam of such length would also be unfeasible from the perspective of a hand-made fabrication process.

**Table 16:** RJS Method data for assessment of the sandwich beam, based on the mean values

	Experimental flexural modulus (MPa)	Theoretical flexural modulus (GPa)	Pure bending amount (%)	Flexural stiffness (N∙mm^2^)	Shear stiffness (N)	Core shear modulus (MPa)	Support span to promote 95% of pure bending (mm)
Affected by balsa’s low transverse stiffness	159.9	1.370	11.67	1.618 × 10^9^	2.207 × 10^4^	6.335	3617
FEA estimation by nullifying the balsa orthotropic effect	366.1		26.72		5.537 × 10^4^	15.89	-
RJS factors							
$\overline{\text{RJS} }$ factor for core rigidity relevance					33.93%		
${\text{RJS}}^{*}$ factor for shear rigidity relevance	Affected by balsa’s low transverse stiffness				0.06		
	FEA estimation by nullifying the balsa orthotropic effect				0.14		

By employing the FEA model for the four-point bending of the sandwich beam (which validation is shown further in Sect. 3.3.3), the orthotropic effect of balsa wood can be nullified by considering the skins as an isotropic material with an elastic modulus of 2.50 GPa (the modulus along the balsa wood grain direction, as previously depicted in Table 8). As indicated in Table 16, eliminating this orthotropic effect would result in an experimental flexural modulus and a pure bending amount approximately 2.3 times higher (~ 366 MPa and ~ 27%, respectively). This adjustment enables the application of the RJS equations to accurately estimate the actual shear stiffness and core shear modulus of the sandwich beam, denoted as 5.537 × 10^4^ N and ~ 16 MPa, respectively.

Still, in Table 16, the $\overline{\text{RJS} }$ factor for core rigidity relevance is ~ 34%, meaning that, under pure bending, the trussed beam as the core contributes to about one-third of the flexural modulus of the sandwich beam made with balsa wood skins. The ${\text{RJS}}^{*}$ factor for shear rigidity relevance is 0.14, classifying the sandwich beam as having a negligible rigidity core when combining the trussed beam with balsa wood skins. It is worth noting that, although balsa wood may not initially appear suitable for achieving the desired stiffness in the linear regime of the sandwich beam under bending through short spans, this sandwich configuration has demonstrated promising performance in the nonlinear regime, as previously detailed in the text (Sect. 3.3.1); by achieving a higher maximum load due to improved load distribution, which helps prevent pullout failures.

Table 17 illustrates how modifying the skins of the sandwich beam while maintaining the same trussed beam core could influence the elastic behaviour of the structure, based on the RJS equations. These modifications are proposed to eliminate the orthotropic effect of balsa wood and enhance the experimental flexural modulus of the sandwich beam while maintaining the same four-point bending configuration (315 mm support span and 105 mm load span). The first modification involves replacing the ~ 5 mm balsa wood skins by ~ 3 mm thick polymeric laminates reinforced with randomly oriented coir fibres, which exhibit a flexural modulus of approximately 2.6 GPa [83] (similar to the grain-direction modulus of balsa, 2.5 GPa), and a density of ~ 1 g/cm^3^ [84]. Given the random fibre orientation, this coir composite laminate can be assumed to be quasi-isotropic. The modified sandwich beam would possess an estimated experimental flexural modulus of ~ 378 MPa, representing a 136% increase compared to the configuration with balsa wood skins. Due to the higher density of the coir laminate, the total mass of the sandwich beam would increase by approximately 100 g compared to the initial mass of 300 g, even despite the lower thickness of the coir laminate. Consequently, the overall density of the sandwich beam with coir skins would be 42% higher. However, the specific flexural modulus would be 66% greater. In other words, replacing balsa wood with coir laminates may not be ideal if the total mass of the structure is a critical constraint. Nevertheless, this substitution could be highly beneficial if superior experimental or specific flexural moduli are required.

**Table 17:** RJS Method data prediction for the sandwich configuration of the trussed beam core with alternative skins

Skin material, thickness (each skin), and elastic modulus	Predicted experimental flexural modulus^∆^ (MPa)	Theoretical flexural modulus (GPa)	Pure bending amount^∆^ (%)	Flexural stiffness (N∙mm^2^)	Shear stiffness (N)	Support span to promote 95% of pure bending (mm)
Coir-fibre composite laminate 3 mm ~ 2.6 GPa, [83]	377.8 (+ 136% ^†^)	1.135	33.29	1.088 × 10^9^	5.140 × 10^4^	1944
Aluminium 0.5 mm ~ 69 GPa, [85]	581.1 (+ 263% ^†^)	4.269	13.61	3.149 × 10^9^	4.697 × 10^4^	3459
RJS factors						
		$\overline{\text{RJS} }$ factor for core rigidity relevance		${\text{RJS}}^{*}$ factor for shear rigidity relevance		
Coir-fibre composite laminate 3 mm ~ 2.6 GPa, [83]		60.43%		0.17		
Aluminium 0.5 mm ~ 69 GPa, [85]		14.96%		0.04		
Estimated equivalent density for the sandwich beams (compared to 0.2141 g/cm^3^ of the sandwich beam made with balsa wood skins)						
Coir-fibre composite laminate 3 mm ~ 1.0 g/cm^3^, [84]		Sandwich beam with mass of ~ 399.2 g and equivalent volume of ~ 1310 cm^3^ → ~ 0.3047 g/cm^3^ (+ 42% ^‡^) for the equivalent density → ~ 1240 MPa∙cm^3^/g (+ 66% ^§^) for the specific flexural modulus				
Aluminium 0.5 mm ~ 2.7 g/cm^3^, [85]		Sandwich beam with mass of ~ 326.8 g and equivalent volume of ~ 1200 cm^3^ → ~ 0.2723 g/cm^3^ (+ 27% ^‡^) for the equivalent density → ~ 2134 MPa∙cm^3^/g (+ 186% ^§^) for the specific flexural modulus				

^∆^Four-point bending with support and load span lengths of 315 mm and 105 mm, respectively. ^†^ Compared to 159.9 MPa of the sandwich beam made with balsa wood skins. ^‡^ Compared to 0.2141 g/cm^3^ of the sandwich beam made with balsa wood skins. ^§^ Compared to 746.7 MPa∙cm^3^/g of the sandwich beam made with balsa wood skins

Still, in Table 17, the estimates for the second modification are presented: employing 0.5 mm thick aluminium skins with an elastic modulus of 69 GPa and a density of 2.7 g/cm^3^ [85]. The experimental flexural modulus of the new sandwich beam would be ~ 581 MPa, representing a 263% increase compared to the sandwich beam with balsa wood skins. Notably, this flexural modulus is equivalent to that of the trussed beam (previously depicted in Table 14). In summary, these aluminium skins would enable the sandwich beam to achieve the same stiffness as the trussed beam while also providing the previously mentioned (Sect. 3.3.1) increase in maximum load capacity due to the absence of pullout failures. Despite aluminium’s higher density, the extremely thin 0.5 mm skins would lead to only a 27% increase in the overall density of the sandwich beam while improving the specific flexural modulus by 186%. However, it is important to highlight that balsa wood surpasses aluminium in terms of sustainability for an eco-friendly design.

#### FEA results

**Fig. 22:**
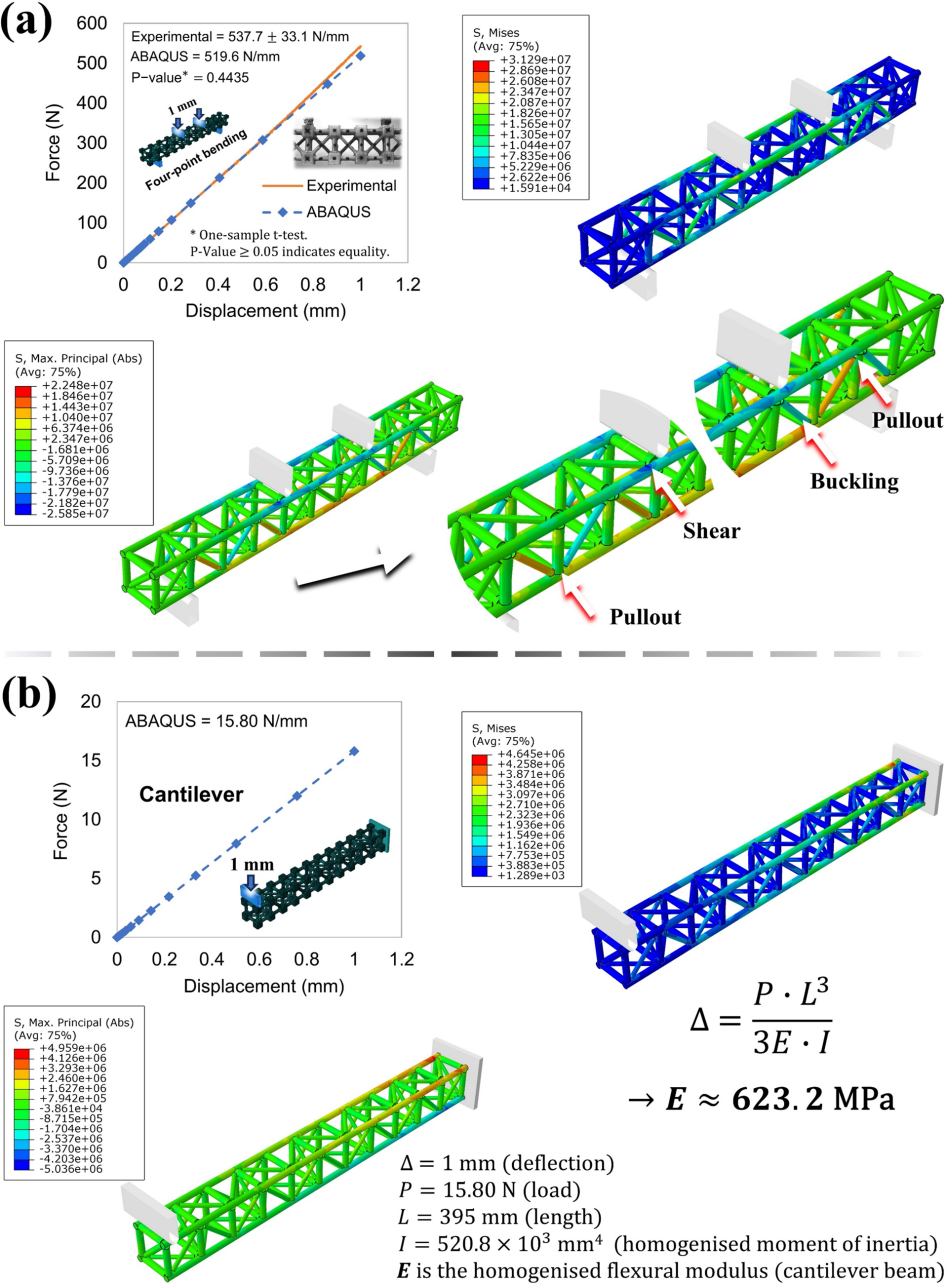
FEA insights on the trussed beam: **a** validation of the model under four-point bending and evaluation of stress distribution in the rods; and **b** results on the cantilever bending assessment

Figure 22a presents an experimental–numerical comparative analysis of the force–displacement curves, confirming the validity of the FEA model for bending of the trussed beam, supported by a P-value exceeding 0.05 through the one-sample t-test. Additionally, the von Mises stress and maximum principal stress distributions highlight critical stress concentrations in regions prone to damage, consistent with observations previously depicted in Fig. 20. Building on the validated FEA model, Fig. 22b illustrates the load corresponding to a 1 mm displacement of the trussed beam in a cantilever configuration, yielding a force of ~ 16 N. By applying the analytical equations covered in [82], the homogenised flexural modulus of the cantilever trussed beam is estimated to be approximately 623 MPa.

**Fig. 23:**
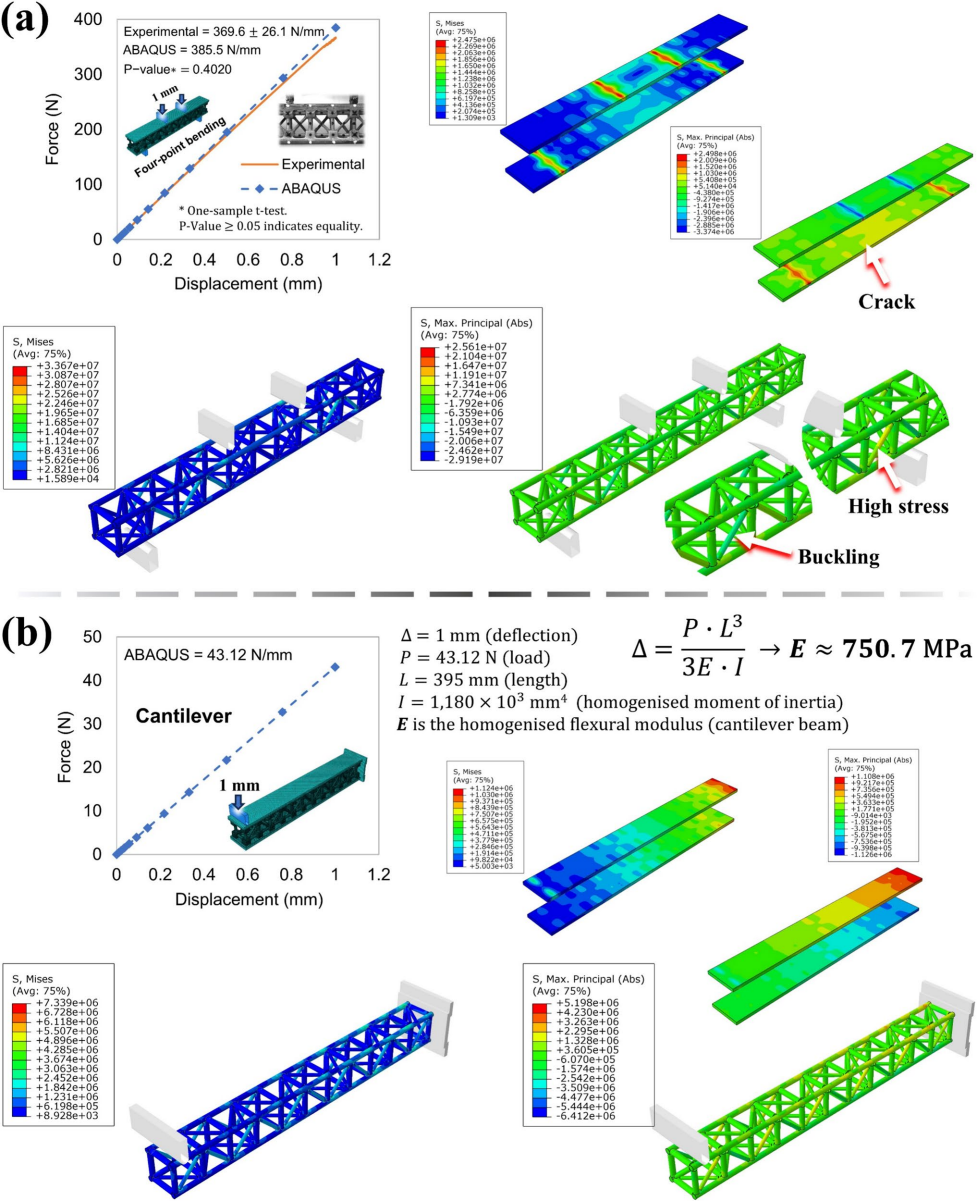
FEA insights on the sandwich beam: **a** validation of the model under four-point bending and evaluation of stress distribution in the rods; and **b** results on the cantilever bending assessment

Figure 23a provides an experimental–numerical comparison of the force–displacement curves, also corroborating the accuracy of the FEA model for the bending of the sandwich beam by a statistical comparison. The von Mises stress and maximum principal stress distributions identify critical stress concentrations in regions prone to damage, aligning with the patterns depicted in Fig. 21: crushing and cracking in the skins and buckling in the auxiliary member on the third cell in one of the sides of the beam, concomitantly to a high tensile stress in the auxiliary member on the opposite side, where the crack propagates through the skins, adhesive layer, and joint. Expanding on the validated FEA model, Fig. 23b depicts the load associated with a 1 mm displacement of the trussed beam in a cantilever configuration, resulting in a force of approximately 4 N. Using the analytical equations outlined in [82], the homogenised flexural modulus of the cantilever trussed beam is calculated to be around 751 MPa.

It is noteworthy that the homogenised flexural modulus of the trussed beam in the cantilever configuration is reasonably consistent with the experimental modulus obtained under four-point bending (~ 623 MPa compared to ~ 587 MPa). However, for the sandwich beam, the modulus estimated in the cantilever configuration is nearly five times greater than the experimental value under four-point bending (~ 751 MPa compared to ~ 160 MPa). Note that the same flexural modulus of the sandwich beam under the cantilever configuration represents approximately 55% of the modulus expected under pure bending (~ 1.37 GPa, as reported in Table 16), while the experimental modulus under four-point bending is about 12% of the pure bending value. This discrepancy is consistent with the fact that the cantilever configuration involves a greater bending length, which may reduce the shear effects in the sandwich beam, thereby yielding a higher homogenised flexural modulus. Furthermore, unlike the four-point bending configuration, in which balsa wood undergoes crushing at four distinct points (two at the support tool and two at the loading tool), the cantilever configuration has only one crushing point: at the loading application site. This also mitigates the effect of balsa crushing on the overall stiffness reduction of the sandwich beam.

### A brief comparison with data from open literature

Table 18 provides a performance comparison, by specific load capacity, between the biobased structures (cell and truss) developed in this work and data available from open literature related to synthetic carbon-fibre-based structures designed for high-performance applications. The trussed cell is compared with carbon composite truss structures produced via additive manufacturing by Poddar et al. [6], previously depicted in Figs. 1a_3_. One of the proposed configurations supports a compressive load of 21,773 N and has a total mass of 571 g. The trussed beam is compared to the carbon fibre trusses manufactured through a filament winding process, as described by Hunt et al. [5], featuring straight-flat shear web configurations and a triangular cell height of 66 mm, as previously depicted in Fig. 1a_2_. This truss configuration from Hunt et al [5]. is tested under three-point bending with a support span of 396 mm and has a mass of 42.2 g per meter of length. In terms of specific maximum load under compression, the biobased metastructure cell outperforms its synthetic counterpart by a significant 447%. The trussed beam, however, exhibits a 30% lower performance under bending when compared to the carbon-composite trusses, though it offers the advantage of being fully composed of renewable materials.

**Table 18:** Specific performance comparison between the here-proposed sustainable trussed structures and those reported in the literature

Here-proposed sustainable structures	Literature report	Property for comparison	Sustainable structures (property)	Literature report (property)	Sustainable structures’ performance**
Metastructure cell	Poddar *et al* [6]*.* Figure 1 (a_3_)	Specific maximum load	208.5 N/g	38.13 N/g	+ 447%
Trussed beam	Hunt *et al* [5] Figure 1 (a_2_)	Specific maximum bending moment*	694.9 N∙mm/g	996.4 N∙mm/g	− 30%

^*^Normalised by the mass of material within the support span length in bending

^**^ $$100\times \left|\text{sustainable structures}-{\text{literature}}\right|/({\text{literature}})$$

**Fig. 24:**
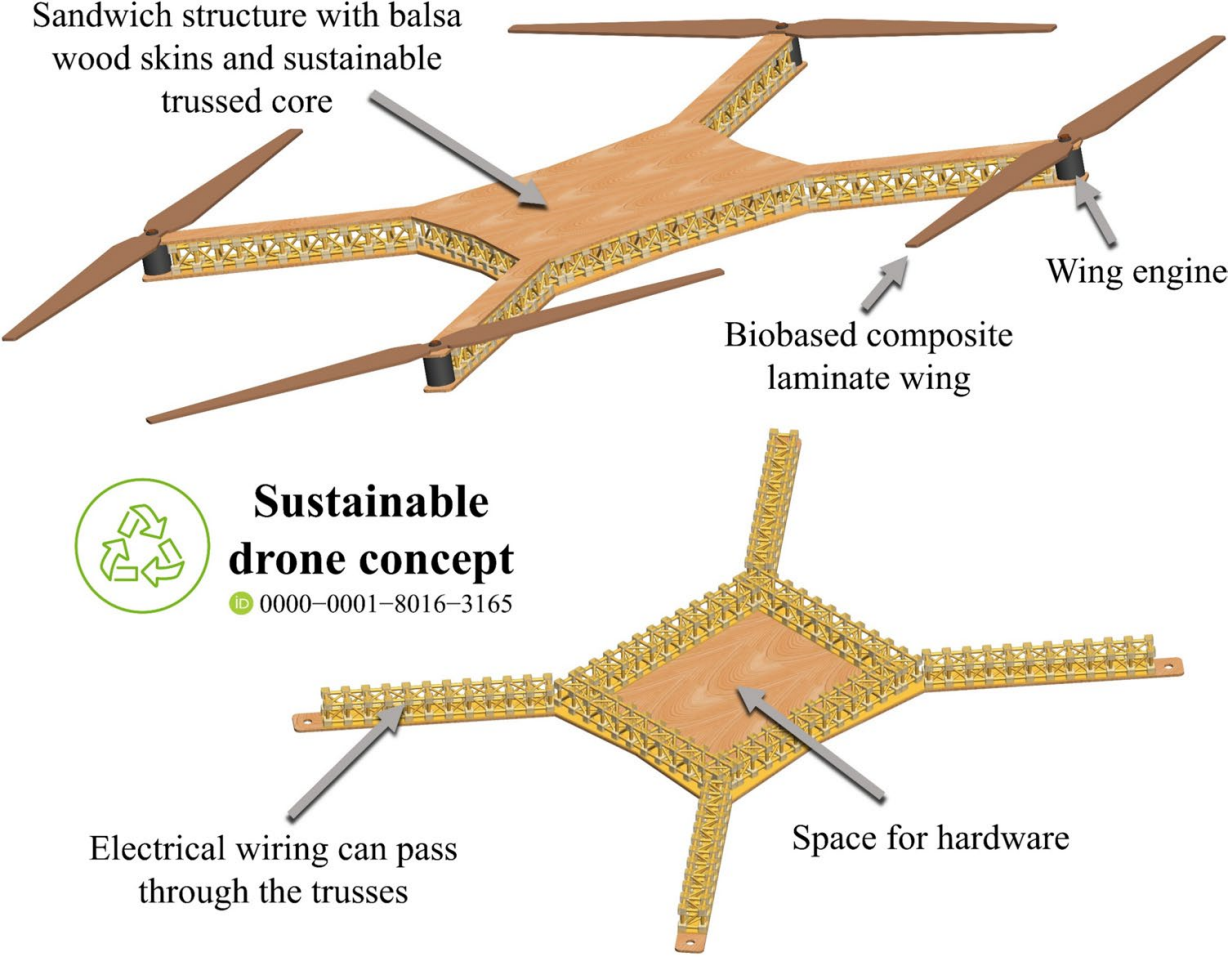
Conceptual drawing of a drone featuring a structure composed of sandwich beams with balsa wood skins and a trussed core entirely fabricated from bio-based materials

These characteristics of high performance per weight of material, coupled with sustainability, make the bio-based trussed structures promising for applications in lightweight transport design, such as the concept of a drone that could be produced using sandwich structures with the trussed beam as core material and balsa wood skins—Fig. 24. In the context of drone applications, the assessment of the impact performance [86–91] of the proposed sandwich beam becomes a critical avenue for future research, particularly to ensure structural integrity and resilience during operations or accidental collisions. It is worth noting that, although the castor oil polymer as a coating material has been used here solely for the bamboo rods, it could also be applied to the balsa skins to prevent water absorption in the drone structure. Furthermore, the current dependence on meticulous and labour-intensive assembly, coupled with the complex nature of its production and protracted development timescales, may likely contribute to the drone’s elevated cost relative to readily available alternatives. However, as fabrication techniques advance and automation enhances efficiency, these constraints may be progressively abated, so that the conceptual design proposed here can ultimately achieve a trade-off balance between cost, performance, and an eco-friendly approach.

## Conclusions

This study successfully demonstrates the design, fabrication, and testing of a metastructure trussed cell composed of bamboo rods and bi-phasic plant-based polymers, offering a sustainable solution for structural applications by proposing the modular assembly of trussed and sandwich beams. The experimental results show that the metastructure trussed cell (design depicted in Fig. 4), with a mass of approximately 30 g, supports up to 700 kg under compression, with a displacement of ~ 2 mm, a rotation of 4°, and an energy absorption of ~ 750 μJ/mm^3^. Within the elastic regime of the trussed cell, sustained up to a displacement of 1 mm, an equivalent zero Poisson ratio is demonstrated alongside a force–displacement slope of ~ 4,200 N/mm. The trussed and sandwich beams (designs illustrated in Fig. 5) exhibit equivalent densities of ~ 0.19 g/cm^3^ and ~ 0.21 g/cm^3^, respectively, and perform exceptionally well under bending loads, with the trussed beam supporting nearly 2,000 N (maximum bending moment of ~ 103 kN∙mm) and the sandwich beam achieving a loading capacity of nearly 3600 N (maximum bending moment of ~ 188 kN∙mm). The modulus of toughness (energy absorbed under four-point bending prior to failure) is ~ 158 μJ/mm^3^ for the trussed beam and ~ 196 μJ/mm^3^ for the sandwich beam. The finite element analysis (FEA) models were successfully validated against the experimental data, demonstrating their accuracy in simulating the elastic behaviour of the structures. These validated models were effectively employed to investigate additional loading configurations, including torsion of the trussed cell and cantilever bending of the beams: the trussed cell depicts a response torque of ~ 7300 N∙mm for 1° of angle twist, while the trussed and sandwich beams have a homogenised flexural modulus of ~ 623 MPa and ~ 751 MPa, respectively. The findings provide critical insights into the performance and potential applications of these sustainable materials in a variety of structural contexts, validating the potential of bamboo-based metastructures as renewable, high-strength, and lightweight alternatives for load-bearing components. Overall, the proposed bamboo-based metastructure trussed cell and its modular integration into trussed and sandwich beams represent a promising direction for enhancing sustainability and structural performance in fields ranging from civil construction to aerospace engineering.

Future research efforts will focus on the dynamic evaluation of the proposed structures, as vibration transmissibility and modal analysis tests, encompassing both experimental and numerical approaches. Further investigations may also explore attempts to optimise the geometry and arrangement of the trussed cell and its elements (rods and joints). The provision of a new joint design would also enhance pullout strength, addressing the critical failure mode of the trussed beam under bending. Further research could explore the impact of bamboo moisture content and castor oil resin curing temperature on bonding joints to rods, as well as the use of different raw materials or rod cross-sections, such as prismatic shapes. The possibility of modifying the positioning of the auxiliary members to nullify the metabehaviour of rotation under compression can also be assessed. While this modification may tend to counteract the rotational behaviour of the metastructure, it could potentially contribute to increased compression rigidity and strength. Another promising topic of study is the investigation of the potential scale effect on the beams, i.e., how their length (number of cells) may affect the mechanical properties under bending. Alternative skins (such as thin aluminium laminae, which have demonstrated promising performance based on initial analytical estimations) and adhesive configurations for manufacturing and testing the sandwich beam, by employing the same here-proposed trussed beam, build another pillar for future research.

As fabrication technologies evolve, the implementation of automated or semi-automated assembly techniques could significantly improve scalability and reduce production costs while maintaining structural performance and sustainability. Future investigations should therefore prioritise the development and optimisation of such processes. Additionally, the long-term durability of the proposed structures must be addressed. Given the biodegradable nature of bamboo and balsa wood, and the potential for biobased polymers to degrade under UV exposure, high humidity, or biological activity, it is crucial to assess the ageing behaviour and structural integrity of the raw materials and assembled components over time. These evaluations are essential for ensuring the reliability and long-term applicability of the structures in real-world scenarios.

## Data Availability

Data can be made available on request to R.J.S., the corresponding author at CERN.

## Appendix: literature report

Developed by Elsevier Publishing, the Engineering Village® platform is consolidated by providing access to 12 databases of indexed documents from a wide range of sources. Detailed searches are possible through a combination of keywords (named string) relevant to specific topics. An initial search correlated to the presence of the terms “bamboo and (truss or lattice)” in all fields returns 202 records of indexed documents in the English language. Sequentially, the “controlled vocabulary” tool can be used to standardise the way articles are indexed, which allows consistent and accurate search related to the topics of interest (bamboo or trusses); and the number of records is reduced to 104 documents. Finally, a classification filter can be applied through the selection of the following topics: Materials Science, Mechanics, Strength of Building Materials, Structural Design, and Structural Members and Shapes. The final query yields 54 records, comprising 37 journal articles, with the remainder consisting of conference papers, articles in press, or preprints. The strings used at each stage of the search process are shown in Table 19.

**Table 19:** Strings for the “expert search” field in the Engineering Village® platform

String	Records
((((((((bamboo) WN ALL) AND ((truss) OR (lattice) WN ALL)))))) AND ({english} WN LA))	202
(((((((((bamboo) WN ALL) AND ((truss) OR (lattice) WN ALL)))))) AND ({english} WN LA)) AND (({bamboo} OR {trusses}) WN CV))	104
((((((((((((bamboo) WN ALL) AND ((truss) OR (lattice) WN ALL)))))) AND ({english} WN LA)) AND (({bamboo} OR {trusses}) WN CV)))) AND (({408.2} OR {951} OR {408.1} OR {422} OR {931.1}) WN CL))	54

The records reported refer to a systematic review performed on the date of February 28, 2025 (the submission date of the first version of this manuscript)

## Footnotes

1 RJS is an acronym derived from the initials of the author’s name, Rodrigo José da Silva.

2 It is worth noting that the concept of yield and the 0.2% offset method are traditionally associated with metallic materials. Here, and in [34], they are applied as pseudo-yield concepts, serving primarily as an analytical approach to determine the linearity limit within the elastic regime of the loading curve.

3 For all graph plots presented in this manuscript, the curve shown corresponds to the specimen whose mechanical properties are most representative (typical) of the mean values reported in the associated tables, which include both the mean and standard deviation.
